# Supplementary material for: Oocyte-Specific Homeobox 1, Obox1, Facilitates Reprogramming by Promoting Mesenchymal-to-Epithelial Transition and Mitigating Cell Hyperproliferation
Source: Stem Cell Reports. 2017 Oct 12;9(5):1692–705. doi: 10.1016/j.stemcr.2017.09.012 (PMC5853649; doi:10.1016/j.stemcr.2017.09.012)
Supplement: Document S1. Supplemental Experimental Procedures, Figures S1–S5, and Tables S3 and S4 [file mmc1.pdf]

Stem Cell Reports, Volume *9*

Supplemental Information

Oocyte-Specific Homeobox 1, *Obox1*, Facilitates Reprogramming by Promoting Mesenchymal-to-Epithelial Transition and Mitigating Cell Hyperproliferation

Li Wu, You Wu, Bing Peng, Zhenzhen Hou, Yu Dong, Kang Chen, Mingyue Guo, Han Li, Xia Chen, Xiaochen Kou, Yanhong Zhao, Yan Bi, Yixuan Wang, Hong Wang, Rongrong Le, Lan Kang, and Shaorong Gao

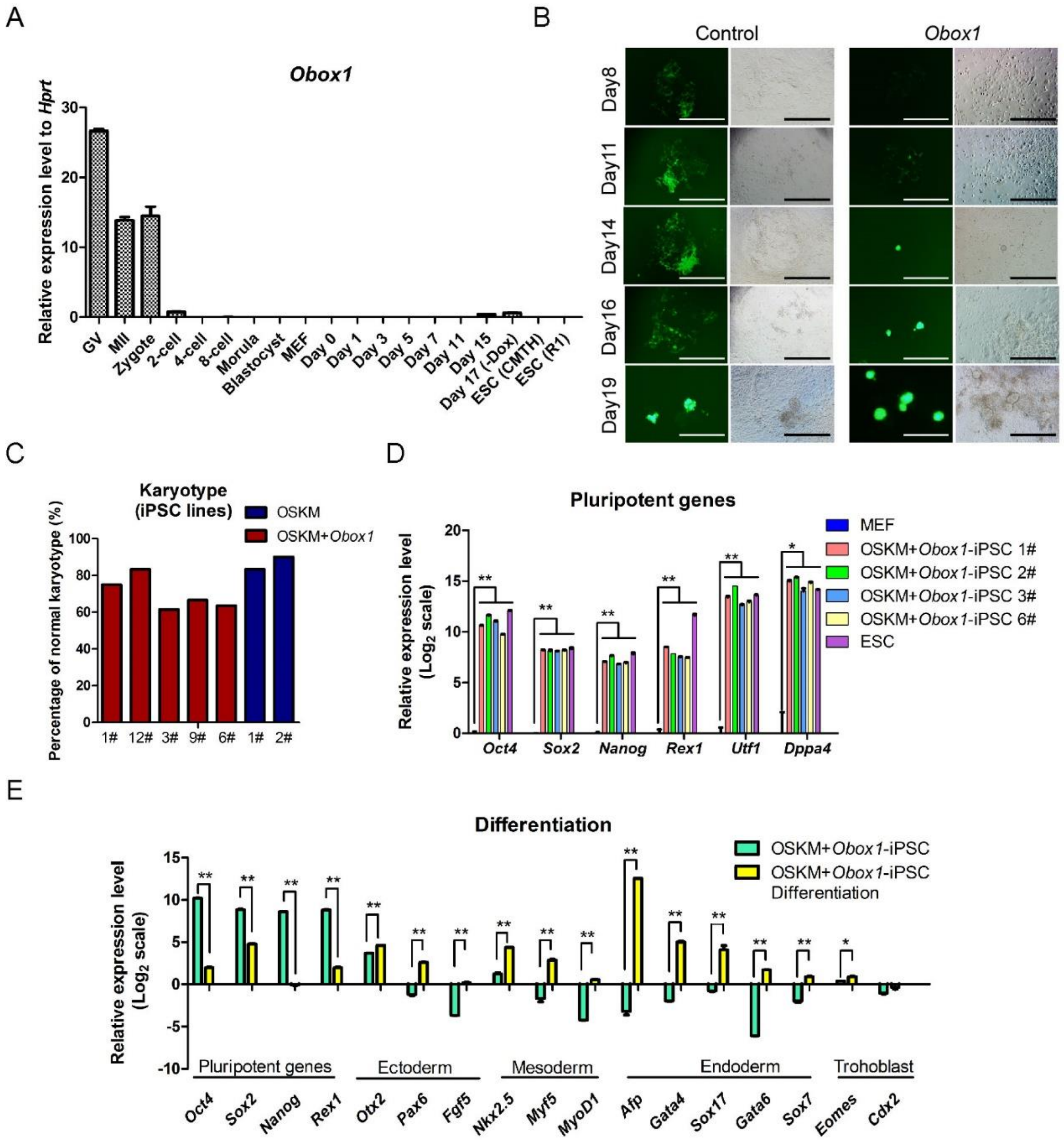

Figure S1. Exogenous Expression of *Obox1* Can Promote iPSC Generation. Related to Figure 1.

(A) Expression patterns of *Obox1* in mouse preimplantation embryos and reprogramming process by qRT-PCR analysis. Relative mRNA level was normalized to hypoxanthine-guanine phosphoribosyltransferase (*Hprt*) (MEF as control).

(B) Morphology of reprogrammable cells at indicated time points. Scale bars, 600 μm.

(C) Karyotype analysis of OSKM+*Obox1*- and OSKM-iPSC lines.

(D) qRT-PCR analysis reveals pluripotent gene expression in OSKM+*Obox1*-iPSCs. Relative mRNA expression was represented relative to expression in MEF as a control.

(E) qRT-PCR analysis shows the upregulation of markers for the three germ layers during in vitro differentiation of OSKM+*Obox1*-iPSCs. Relative mRNA expression was represented relative to expression in the undifferentiated iPSCs as a control.

Data are represented as the mean ± SEM ($n = 3$); * $p < 0.05$, ** $p < 0.01$ by Student's t test for comparison.

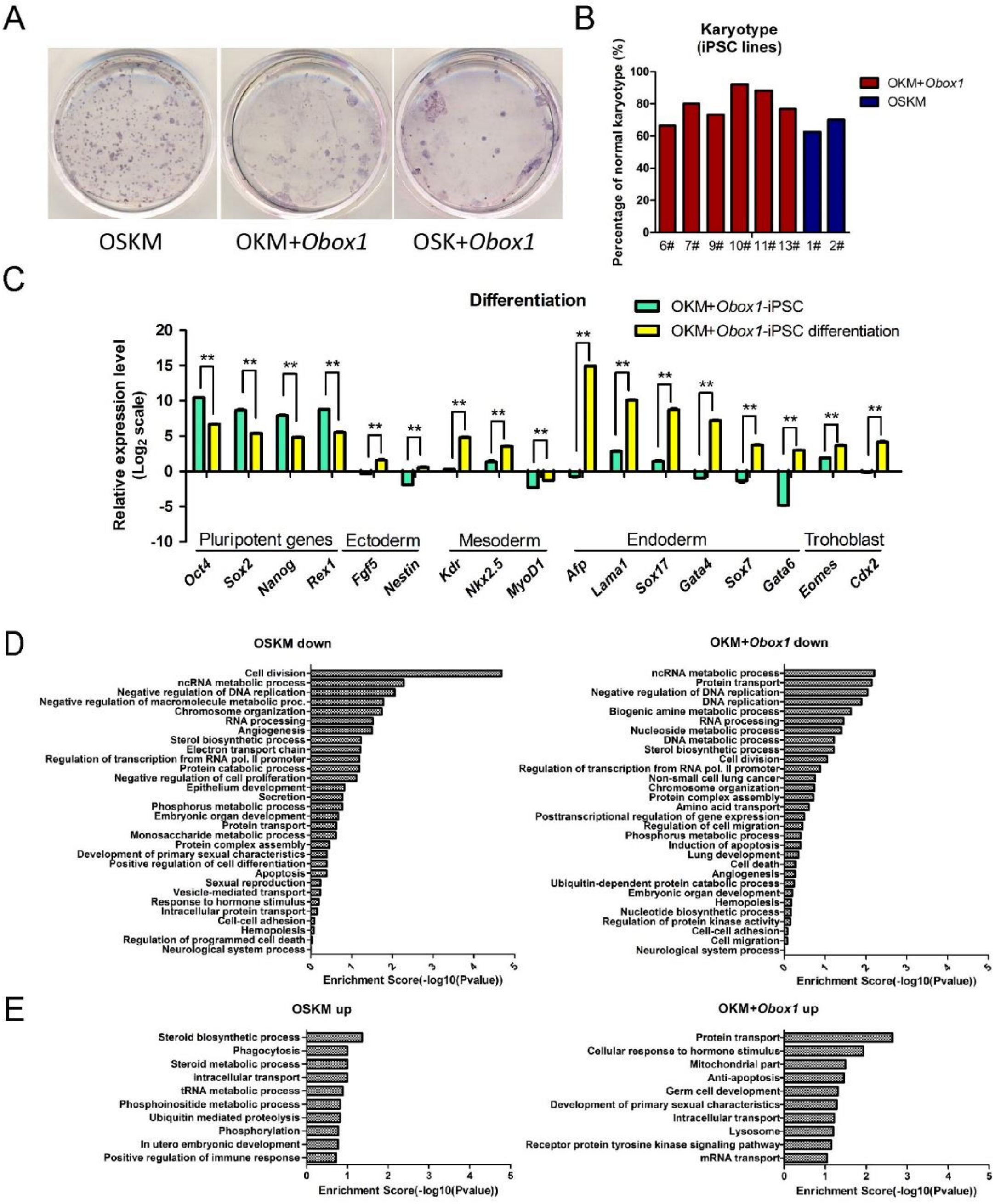

Figure S2. Characterization of OKM+*Obox1*-iPSCs and Genome-wide Analysis of OSKM and OKM+*Obox1* in Reprogramming. Related to Figure 2.

(A) The iPSCs colonies were detected by AP staining.

(B) Karyotype analysis of OSKM- and OKM+*Obox1*-iPSC lines.

(C) qRT-PCR analysis shows the upregulation of markers for the three germ layers during in vitro differentiation of OKM+*Obox1*-iPSCs. Relative mRNA expression is represented relative to expression in the undifferentiated iPSCs as a control. Data are represented as the mean ± SEM (n = 3); * $p < 0.05$, ** $p < 0.01$ by Student's t test for comparison.

(D) Gene ontology analysis of different expression genes downregulated in the samples of OSKM and OKM+*Obox1*, comparing with OKM+Vector, on reprogramming day 3.

(E) Gene ontology analysis of different expression genes upregulated in the samples of OSKM and OKM+*Obox1*, comparing with OKM+Vector, on reprogramming day 3.

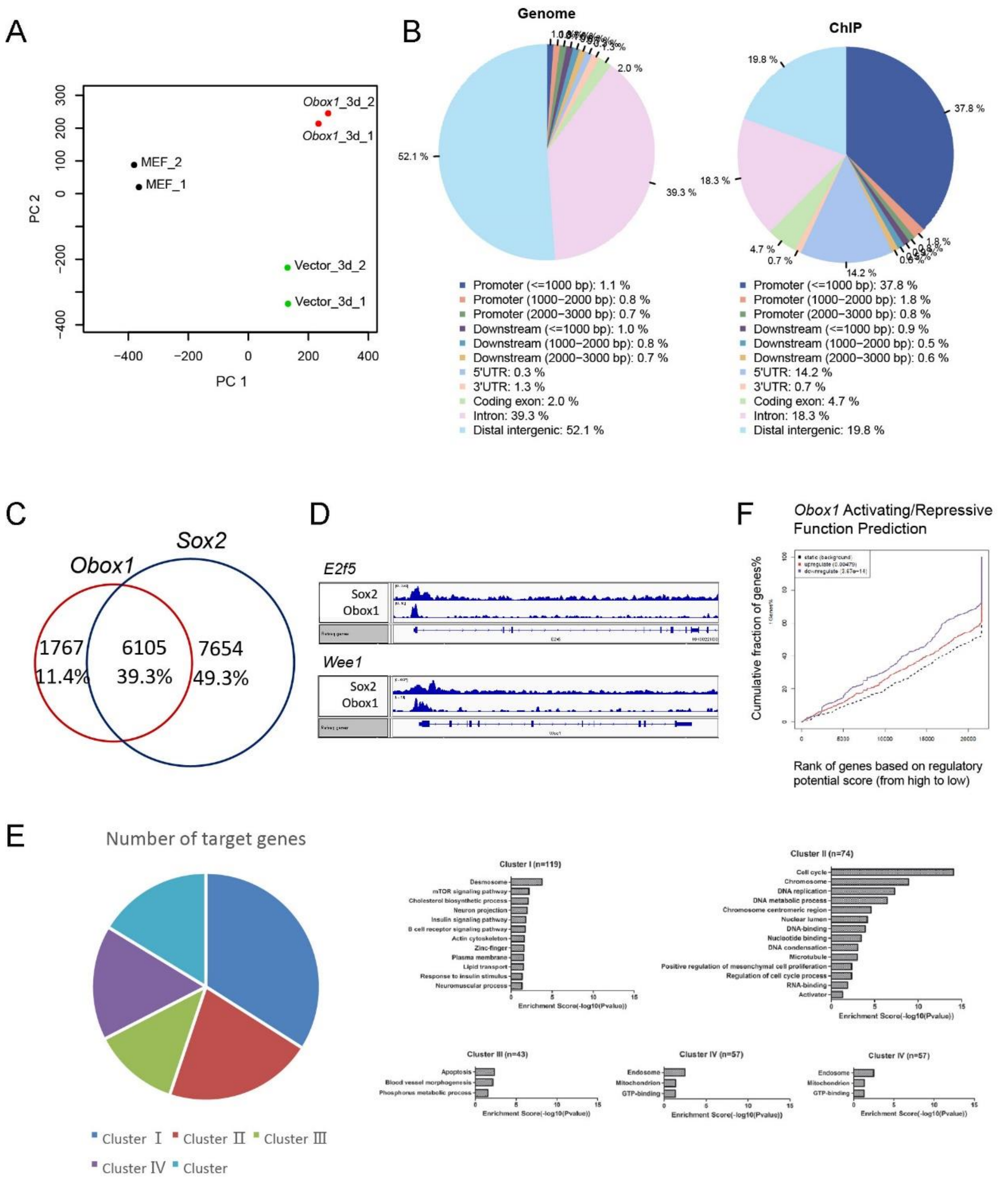

Figure S3. Genome-wide Analysis of the Effects of *Obox1* Overexpression on Somatic Reprogramming. Related to Figure 4.

(A) Principal component analysis (PCA) of global expression profiles of indicated samples. Contributions of each cell (dots) to the first two principal components (PC1 and PC2).

(B) Pie charts show genome distribution of *Obox1* binding sites on reprogramming day 3.

(C) Venn diagram shows the overlap of target genes between *Obox1* in OSKM+*Obox1* samples and *Sox2* in OSKM previously reported.

(D) ChIP density profiles of *Obox1* and *Sox2* at cell cycle-related gene promoters.

(E) Pie chart shows different expression genes between samples (*Obox1* and Vector) which are also targeted

by *Obox1* through ChIP-seq. These genes were clustered by K-means. Gene ontology analysis of each cluster was shown in the right panel.

(F) Activation or repression function prediction of *Obox1* on reprogramming day3.

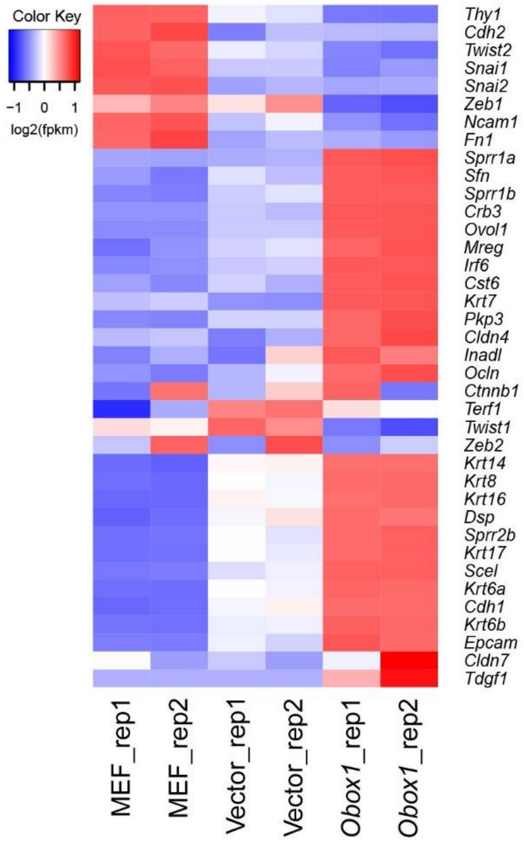

Figure S4. *Obox1* Promotes MET in the Initiation Stage of Reprogramming. Related to Figure 5. Heatmap shows the expression patterns of epithelial and mesenchymal-associated genes. FPKM values were analyzed using Cufflinks and represented in color (Red: upregulated genes; blue: downregulated genes).

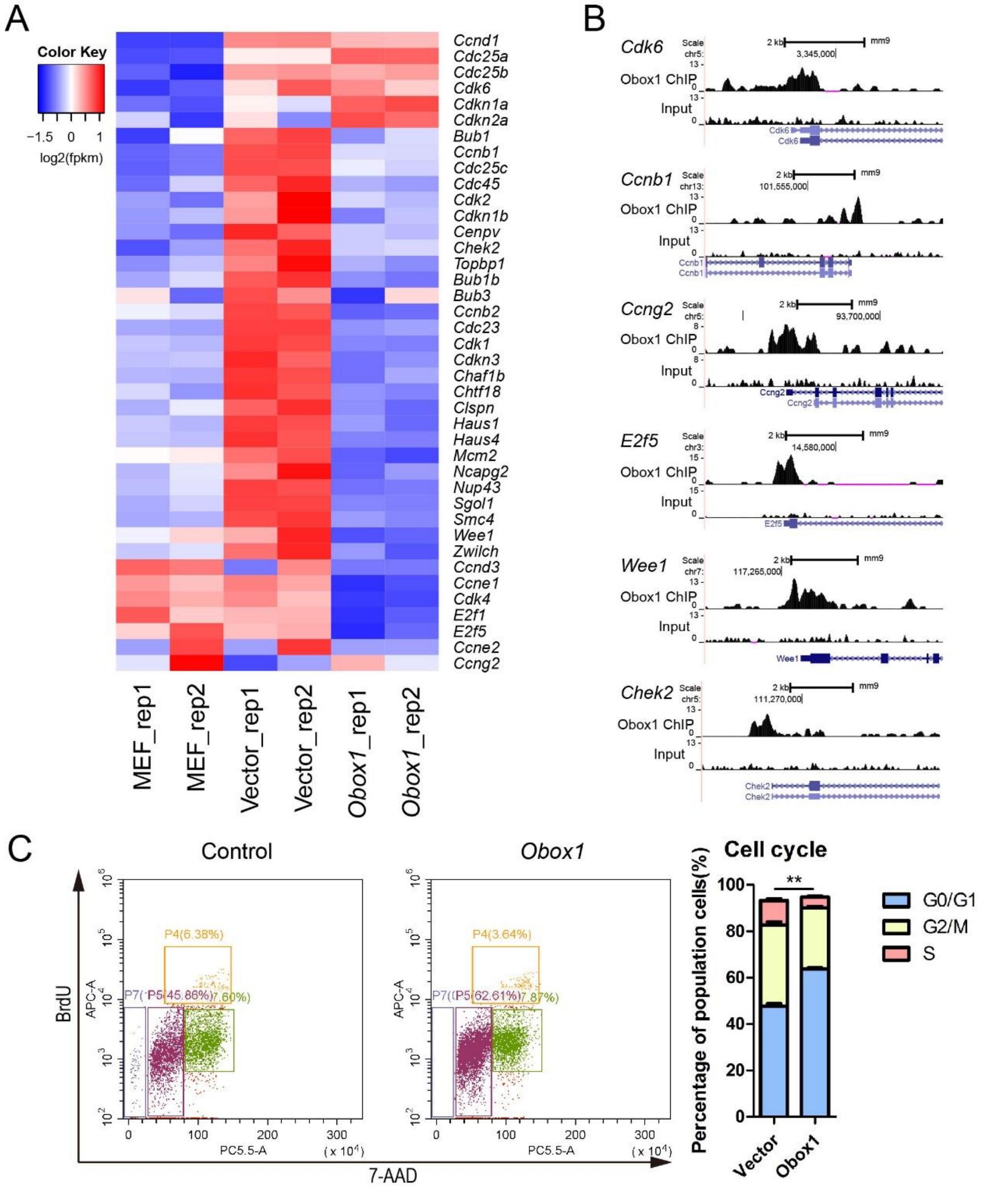

Figure S5. ***Obox1*** **Mitigates Cell Hyper-proliferation by Modulating Cell-cycle-related Genes Expression. Related to Figure 6.**

(A) Heatmap shows the expression patterns of cell cycle-related genes in the indicated samples. FPKM values were analyzed using Cufflinks and represented in color (red: upregulated genes; blue: downregulated genes).

(B) ChIP density profiles of *Obox1* at cell-cycle-related gene promoters.

(C) Cell-cycle analysis of the reprogramming cells on day 2 post-induction by FACS. Reprogrammable fibroblasts were transfected with *Obox1* or empty vector (control) and were labeled with 5-bromodeoxyuridine (BrdU) and 7-AAD after 2-days induction by Dox. Data are represented as the mean ± SEM (n = 3); $^{**}p < 0.01$ by Student's t test for comparison and empty vector in each phase as control.

Table S1. Primer Sequences Used in this Paper. Related to Figure 1. (see attached the excel file named "Table S1 primers").

Table S2. Different Expression Genes with or without *Obox1* on Reprogramming Day 3. Related to Figure 4. (see attached the excel file named "Table S2 DEGs")
Different expression genes between samples (Vector and *Obox1*) on reprogramming day 3 were grouped into five clusters by K-means clustering based on RNA-seq data. The potential targets of *Obox1* based on the ChIP-seq data are presented as 1 in the binding column.

Table S3. Epithelial and Mesenchymal Genes Regulated by *Obox1* at Early Stage during Reprogramming in RNA-seq Data. Related to Figure 5.

Gene Name	MEF_rep1	MEF_rep2	Vector_rep1	Vector_rep2	*Obox1* _rep1	*Obox1* _rep2	km$cluster
Thy1	1.19478961	1.17627334	-0.08987027	-0.21514261	-1.02267208	-1.04337799	1
cdh2	1.1518509	1.38002649	-0.98388087	-0.48171426	-0.52271892	-0.54356335	1
Twist2	1.28280276	1.13412558	-0.13649092	-0.30950535	-0.90974834	-1.06118373	1
Snai1	1.33111583	1.19484816	-0.41882224	-0.40705972	-0.93435999	-0.76572204	1
Snai2	1.26898814	1.30970161	-0.69786155	-0.5555709	-0.67886999	-0.64638731	1
Zeb1	0.53447952	0.92126836	0.19763562	0.84001313	-1.1679552	-1.32544143	1
Ncam1	1.15524151	1.27416863	-0.44594427	-0.08301379	-0.82715139	-1.07330069	1
Fn1	1.15029952	1.41267184	-0.68570788	-0.50110428	-0.60757672	-0.76858248	1
Sprr1a	-0.66658993	-0.69949865	-0.62509236	-0.58854239	1.26027542	1.3194479	2
SFN	-0.75101009	-1.01051372	-0.24053218	-0.49281824	1.21644024	1.27843399	2
SPRR1B	-0.91067661	-0.97195562	-0.37619676	-0.20749396	1.23014658	1.23617637	2
Crb3	-0.81702459	-0.81702459	-0.4047521	-0.50795579	1.26587727	1.2808798	2
Ovol1	-0.86900487	-0.86518482	-0.39538943	-0.3939873	1.24545531	1.27811112	2
Mreg	-1.06732939	-0.82288821	-0.33864536	-0.22394931	1.15331378	1.29949849	2
Irf6	-0.90115179	-0.84664117	-0.41859092	-0.35226852	1.25736147	1.26129094	2
Cst6	-0.70792666	-0.89895809	-0.32517639	-0.60469395	1.23874204	1.29801305	2
Krt7	-0.48069164	-0.3675408	-0.83103995	-0.85513001	1.25347495	1.28092744	2
Pkp3	-0.90527369	-0.9249384	-0.32495373	-0.33006029	1.1371158	1.34811032	2
Cldn4	-0.52441729	-0.42753272	-0.97163878	-0.60581528	1.13788384	1.39152023	2
Inadl	-0.94768337	-0.60563864	-1.03283367	0.34535136	1.25911607	0.98168825	2
Ocln	-0.81537323	-0.98512755	-0.55791816	-0.09900547	1.11883026	1.33859415	2
Ctnnb1	-1.04312919	1.05639042	-0.5480357	0.37130786	1.16440302	-1.00093641	3
Terf1	-1.60502563	-0.62252579	0.92398174	1.05796195	0.25158087	-0.00597313	3
Twist1	0.25778189	0.10424331	1.15535101	0.85324054	-1.02732958	-1.34328716	3
Zeb2	-0.4248737	1.17554912	-0.86024516	1.34722358	-0.86032226	-0.37733157	3
Krt14	-1.11078979	-1.19050215	0.05862167	0.07977907	1.08104951	1.08184169	4
Krt8	-1.08468652	-1.1257301	0.01296174	-0.06162595	1.11376026	1.14532057	4
Krt16	-1.12113827	-1.12773275	0.07726782	-0.04724189	1.08269297	1.13615212	4
Dsp	-1.18967522	-1.09729687	-0.07458716	0.20257152	1.12072473	1.038263	4
Sprr2b	-1.08132986	-1.04027598	-0.00081845	-0.20301549	1.12088335	1.20455643	4
Krt17	-1.07986303	-1.07481482	0.0108102	-0.15858346	1.11405835	1.18839276	4
Scel	-1.02931643	-0.99810855	-0.25391749	-0.11317974	1.18904691	1.2054753	4
Krt6a	-1.08561696	-1.10124775	0.00079924	-0.09402318	1.15497857	1.12511009	4
CDH1	-1.1423196	-1.10212199	-0.07072346	0.09409541	1.11156943	1.10950021	4
Krt6b	-1.04165463	-1.0630391	-0.12620211	-0.11555294	1.21005579	1.13639298	4
Epcam	-0.97888062	-0.99703633	-0.1142405	-0.32499888	1.28678063	1.1283757	4
Cldn7	0.01816466	-0.73160057	-0.40121976	-0.73162276	-0.0926879	1.93896633	5
Tdgf1	-0.59747418	-0.59747418	-0.59747418	-0.59747418	0.596494	1.79340272	5

Table S4. Cell-cycle-related Genes Regulated by *Obox1* at Early Stage during Reprogramming in RNA-seq Data. Related to Figure 6.

Gene Name	MEF_rep1	MEF_rep2	Vector_rep	Vector_rep	*Obox1* _rep	*Obox1* _rep	km$cluster
Ccnd1	-1.2691359	-1.283439	0.79142408	0.82035271	0.48033135	0.46046673	1
Cdc25a	-1.2017403	-1.1448599	0.11698711	0.12444912	1.059101	1.04606293	1
Cdc25b	-1.0546181	-1.4980622	0.64615635	0.71242016	0.54419463	0.64990919	1
Cdk6	-1.337109	-1.1003249	0.20943429	1.11785049	0.7692668	0.34088234	1
Cdkn1a	-0.9616175	-1.1764443	0.09357923	-0.241192	1.06478083	1.22089378	1
Cdkn2a	-0.3055234	-1.3357983	0.21298791	-0.7819603	1.21185399	0.99844006	1
Bub1	-1.3042323	-0.0138197	1.02925382	1.27666671	-0.7405146	-0.2473539	2
Ccnb1	-1.0400265	-0.8900404	1.19416565	1.25176417	-0.2486945	-0.2671685	2
Cdc25c	-1.0758373	-0.9003883	1.22431	1.190874	-0.1235188	-0.3154395	2
Cdc45	-0.9924902	-0.3218972	1.03179774	1.46292221	-0.5194378	-0.6608947	2
Cdk2	-0.6535664	-0.9520592	0.64237871	1.71678425	-0.2706404	-0.4828971	2
Cdkn1b	-0.6979677	-0.4351792	0.67428546	1.72390639	-0.852496	-0.412549	2
Cenpv	-0.7106052	-0.9729497	1.45492351	1.04475188	-0.348588	-0.4675325	2
Chek2	-1.1594275	-0.6338279	0.9465372	1.47000592	-0.3496982	-0.2735896	2
Topbp1	-0.7556225	-0.4003039	0.80845837	1.65280036	-0.5271847	-0.7781476	2
Bub1b	-0.5937549	-0.2468363	1.10634307	1.40021768	-0.7487379	-0.9172317	3
Bub3	0.18219483	-1.0229312	1.2098031	0.73522645	-1.3603866	0.25609334	3
Ccnb2	-0.1070647	-0.2469067	1.25337942	1.13414975	-1.0531101	-0.9804477	3
Cdc23	-0.5862805	-0.6343451	1.28766304	1.29099221	-0.7382695	-0.6197601	3
Cdk1	-0.3868754	-0.4739167	1.32322433	1.21274425	-0.8157819	-0.8593946	3
Cdkn3	-0.4266378	-0.3821121	1.45402257	1.04854583	-0.9560651	-0.7377533	3
Chaf1b	-0.4897097	-0.513188	1.35615323	1.18525317	-0.9554549	-0.5830538	3
Chtf18	-0.268972	-0.7053869	1.39225698	1.13299915	-0.7236042	-0.827293	3
Clspn	-0.5502371	-0.1361782	1.05072751	1.40488646	-0.7505688	-1.0186299	3
Haus1	-0.3555721	-0.4888558	1.31865408	1.19856779	-0.6631122	-1.0096819	3
Haus4	-0.3857273	-0.4382543	1.38604776	1.13746006	-0.8476166	-0.8519095	3
Mcm2	0.01598365	0.12854553	0.9650325	1.18520867	-1.0630413	-1.231729	3
Ncapg2	-0.4728761	-0.1635253	0.76289004	1.61035993	-1.0501739	-0.6866748	3
Nup43	-0.4555852	-0.1896167	1.28132533	1.1968358	-0.9511737	-0.8817855	3
Sgol1	-0.5573422	-0.3016527	1.2766524	1.25353174	-0.8161601	-0.8550292	3
Smc4	-0.5810727	-0.504778	1.21611933	1.34981804	-0.6649751	-0.8151115	3
Wee1	-0.0992637	0.28404185	0.5510529	1.47095907	-1.1630848	-1.0437054	3
Zwilch	-0.3760704	-0.2139381	0.94812912	1.46433726	-0.6778914	-1.1445664	3
Ccnd3	1.04526873	0.89860162	-0.9097487	0.7854304	-0.9098107	-0.9097413	4
Ccne1	0.68876784	0.41789441	0.85302794	0.58969591	-1.3884482	-1.1609379	4
Cdk4	0.79575972	0.54225309	0.77790114	0.4406684	-1.3300242	-1.2265581	4
E2f1	1.12814613	0.3569501	0.48906453	0.5027206	-1.3807259	-1.0961554	4
E2f5	0.30729495	1.15058281	0.43983491	0.55217282	-1.4279329	-1.0219526	4
Ccne2	-0.6451431	1.2286046	-0.6449047	1.35143575	-0.6451425	-0.6448501	5
Ccng2	-0.1947631	1.68280367	-1.214312	-0.6070192	0.50699211	-0.1737015	5

SUPPLEMENTAL EXPERIMENTAL PROCEDURES

Mice and Cell Culture

The specific pathogen-free mice were housed in the animal facility of Tongji University. All our study procedures were consistent with the Tongji University Guide for the care and use of laboratory animals. Mouse embryonic fibroblasts (MEFs) were derived from 13.5-dpc embryos. MEFs were maintained in DMEM (Life Technologies) medium supplemented with 10% (vol/vol) FBS (Gibco) and 1 mM L-glutamine (Merck Millipore). ESCs and iPSCs were cultured on mitomycin C treated MEFs in ESM containing DMEM (Merck Millipore) supplemented with 15% (v/v) fetal bovine serum (Hyclone, Logan, UT), 1 mM L-glutamine (Merck Millipore), 0.1 mM mercaptoethanol (Merck Millipore), 1% nonessential amino acid stock (Merck Millipore), and 1000 U/mL LIF (Merck Millipore).

Induction of iPSCs

The constructed plasmid (in Fuw-TET-On vector) preparation and iPSCs induction procedure were performed according to a previously reported method (Kang et al., 2009). Plasmids were extracted with Plasmid Mini Kit (Tiangen, China) and EndoFree Plasmid Maxi Kit (Cwbio, China). HEK293T cells were transfected with the plasmids along with the lentivirus packaging plasmids psPAX2 and pMD2G (VigoFect, China). The medium containing virus was collected 48 hours after transfection. For OSKM+*Obox1* system, reprogrammable MEFs were seeded in 12-well plates at a density of 1.2×10^4 cells per well, and for OSKM or OKM+*Obox1* system, OG2-MEFs were seeded in 6-well plates at a density of 3-5×10^4 cells per well, then were infected with virus-containing medium for 10-12 hours. Infected MEFs were cultured in ESM supplemented with 1 μg/mL Dox. The cells were observed and tested at indicated time points during reprogramming. The colonies were cultured in ESM in the absence of doxycycline for 2-3 days before being mechanically picked.

Alkaline Phosphatase Staining

To detect alkaline phosphatase (AP) staining, the cytochemical assay was performed using alkaline phosphatase staining kit (Beyotime, China) according to the manufacturer’s protocol. In briefly, the cells at the end of reprogramming were washed once by DPBS, and fixed by 10% formaldehyde solution for 5 minutes at room temperature. Then, the cells were washed once by deionized water and stained by the reagent provide by the kit.

FACS Analysis

Cells were trypsinized, washed twice with DPBS, resuspended in FACS buffer (PBS + 0.1% BSA) and analyzed *Oct4*-GFP$^+$ population. For analysis and/or sorting of intermediates, cells were stained with 5 μL of PE/Cy7-conjugated antibody against THY1.2 (BioLegend, 140310) and/or APC-conjugated antibody against SSEA1 (BioLegend, 125608) in 100 μL FACS buffer per 10^6 cells. After 30 minutes staining on ice, cells were washed once in FACS buffer and suspended in FACS buffer for sorting by FACS Aria II (BD) or analyzing by CytoFLEX S (Beckman Coulter).

In Vitro Differentiation

For spontaneous differentiation, iPSCs were trypsinized and a total of 5×10^4 cells per drop were cultured in hanging drop for 2 days and transferred to ultra-low cluster plates (Costar, USA) in DMEM (Gibco) supplemented with 15% (v/v) FBS, 1 mM L-glutamine (Merck Millipore), 0.1 mM mercaptoethanol (Merck Millipore), 1% nonessential amino acid stock (Merck Millipore), but without LIF. Five days later,

EBs were collected on gelatin-coated tissue cultured dishes for 21 days. Total RNA of EBs was extracted and analyzed for the markers for three embryonic germ layers using qRT-PCR. The primer sequences are available in the Table S1.

Teratoma Formation

The iPSCs were trypsinized and a total of 2-5×10^6 iPSCs were subcutaneously injected into the groin of SCID mice. The tumors were dissected 4 weeks after the injection and processed for hematoxylin-eosin staining.

Chimeric Mouse Generation

To produce chimeric mice, the appropriate number of iPSCs were trypsinized and aggregated with two ICR 8-cell stage embryos removed the zona pellucida in each pit. After a 2-day culture, the embryos were transplanted into the uterus of pseudo-pregnant mice. The *Oct4*-GFP^+ germ cells were detected in the gonads from E 12.5 embryos.

Immunofluorescence Staining

Immunofluorescence staining was performed as previously described (Gao et al., 2013). Primary antibodies used were anti-OCT4 (Santa Cruz, SC-5279), anti-NANOG (Cosmo Bio, RCAB001P), anti-SSEA1 (Millipore, MAB4301). DNA was labeled with DAPI (1 μg/mL, Merck Millipore). The following fluorochrome conjugated secondary antibodies were used: Alexa Fluor 594 donkey anti-mouse IgG (Thermo Fisher, A21203), Alexa Fluor 594 donkey anti-rabbit IgG (Thermo Fisher, A21207). The stained cells mounted on slides were observed using an LSM 880 microscope (Zeiss, Germany,) with a Plan Neofluar 63×/1.4 Oil DIC objective.

Quantitative real-time PCR (qRT-PCR)

Total RNA was extracted using TRNzol Universal Reagent (Tiangen, Beijing, China) and reverse transcribed using the 5X All-In-One RT MasterMix (ABM, Richmond, Canada). Quantitative reverse-transcription PCR was performed with SYBR®FAST Universal qPCR Kit (KAPA, Wilmington, US) and the ABI7500 Fast Real-time PCR system (Applied Biosystems, Foster City, CA). The reactions were performed in triplicate using 1/10 concentration of the cDNA obtained as described above. Relative mRNA expression is normalized to *Hprt* as an endogenous control using the ΔΔCT or ΔCT method. Primer sequences are available in the Table S1 (Gao et al., 2013; Le et al., 2014; Samavarchi-Tehrani et al., 2010; Takahashi and Yamanaka, 2006; Xiang et al., 2012; Xiong et al., 2016; Yang et al., 2016).

Oocyte Collection and Embryo Culture

Full grown germinal vesicle-intact (GV) oocytes were obtained from 6-8 weeks female C57BL/6j mice after injection of 10 IU pregnant mare serum gonadotropin (PMSG) for 48 hours, and freed of attached cumulus cells as previously described (Wang et al., 2010). Metaphase II (MII) oocytes were collected from the oviducts of the mice after consecutive injection of females at 8–10 weeks of age with PMSG and human chorionic gonadotrophin (hCG) at 44–48 hours intervals, and the adherent cumulus cells were removed by hyaluronidase treatment. The zygotes were collected from the oviducts of the mice that were mated with male mice. Embryos were cultured in CZB medium at 37 ℃ in a humidified atmosphere of 5% CO2 in air (Gao et al., 2003). Embryos normally cleave and develop to blastocysts and were collected at different stages.

Western Blot Analysis

After removal of the feeder cells, pluripotent stem cells were washed once with DPBS and boiled to 98 ℃ for 10 minutes in loading buffer (BioRad) with 5% β-mercaptoethanol (Amersham, CT). Anti α-TUBULIN (Proteintech, 66031-1-Ig) was used as endogenous control and Anti-E-CADHERIN (Abcam, Ab11512), Pan-CYTOKERATIN (Abcam, Ab7753) was used. ECL peroxidase-labeled sheep anti-mouse antibody (GE Healthcare, NA931VS) or goat anti-rat antibody (Santa Cruz, SC-2006) were used as secondary antibodies.

SUPPLEMENTAL REFERENCES

Gao, S., Chung, Y.G., Williams, J.W., Riley, J., Moley, K., and Latham, K.E. (2003). Somatic cell-like features of cloned mouse embryos prepared with cultured myoblast nuclei. Biology of reproduction *69*, 48-56.

Gao, Y., Chen, J., Li, K., Wu, T., Huang, B., Liu, W., Kou, X., Zhang, Y., Huang, H., Jiang, Y., *et al.* (2013). Replacement of Oct4 by Tet1 during iPSC induction reveals an important role of DNA methylation and hydroxymethylation in reprogramming. Cell stem cell *12*, 453-469.

Kang, L., Wang, J., Zhang, Y., Kou, Z., and Gao, S. (2009). iPS cells can support full-term development of tetraploid blastocyst-complemented embryos. Cell stem cell *5*, 135-138.

Le, R., Kou, Z., Jiang, Y., Li, M., Huang, B., Liu, W., Li, H., Kou, X., He, W., Rudolph, K.L., *et al.* (2014). Enhanced telomere rejuvenation in pluripotent cells reprogrammed via nuclear transfer relative to induced pluripotent stem cells. Cell stem cell *14*, 27-39.

Samavarchi-Tehrani, P., Golipour, A., David, L., Sung, H.K., Beyer, T.A., Datti, A., Woltjen, K., Nagy, A., and Wrana, J.L. (2010). Functional genomics reveals a BMP-driven mesenchymal-to-epithelial transition in the initiation of somatic cell reprogramming. Cell stem cell *7*, 64-77.

Takahashi, K., and Yamanaka, S. (2006). Induction of pluripotent stem cells from mouse embryonic and adult fibroblast cultures by defined factors. Cell *126*, 663-676.

Wang, S., Kou, Z., Jing, Z., Zhang, Y., Guo, X., Dong, M., Wilmut, I., and Gao, S. (2010). Proteome of mouse oocytes at different developmental stages. Proceedings of the National Academy of Sciences of the United States of America *107*, 17639-17644.

Xiang, X., Deng, Z., Zhuang, X., Ju, S., Mu, J., Jiang, H., Zhang, L., Yan, J., Miller, D., and Zhang, H.G. (2012). Grhl2 determines the epithelial phenotype of breast cancers and promotes tumor progression. PloS one *7*, e50781.

Xiong, Z., He, W., Chen, J., Han, Z., Sun, X., and Gao, S. (2016). Study of Mouse Androgenetic Haploid Embryonic Stem Cells on Proliferation Ability. Chinese Journal of Cell Biology *38*, 9.

Yang, P., Shen, W.B., Reece, E.A., Chen, X., and Yang, P. (2016). High glucose suppresses embryonic stem cell differentiation into neural lineage cells. Biochemical and biophysical research communications *472*, 306-312.
