# Supplementary material for: Oocyte-Specific Homeobox 1, Obox1, Facilitates Reprogramming by Promoting Mesenchymal-to-Epithelial Transition and Mitigating Cell Hyperproliferation
Source: Stem Cell Reports. 2017 Oct 12;9(5):1692–705. doi: 10.1016/j.stemcr.2017.09.012 (PMC5853649; doi:10.1016/j.stemcr.2017.09.012)
Supplement: Document S2. Article plus Supplemental Information [file mmc4.pdf]

Stem Cell Reports
Article
ISSCR
OPEN ACCESS

Oocyte-Specific Homeobox 1, *Obox1*, Facilitates Reprogramming by Promoting Mesenchymal-to-Epithelial Transition and Mitigating Cell Hyperproliferation

Li Wu,[1,3,4] You Wu,[1] Bing Peng,[1] Zhenzhen Hou,[1] Yu Dong,[1] Kang Chen,[2] Mingyue Guo,[1] Han Li,[1] Xia Chen,[1] Xiaochen Kou,[1] Yanhong Zhao,[1] Yan Bi,[1] Yixuan Wang,[1] Hong Wang,[1] Rongrong Le,[1,*] Lan Kang,[1,2,*] and Shaorong Gao[1,*]

[1]Clinical and Translational Research Center of Shanghai First Maternity & Infant Hospital, School of Life Sciences and Technology, Tongji University, Shanghai 200092, China
[2]Institute of Cancer Stem Cell, Dalian Medical University, Dalian 116044, China
[3]Institute of Biophysics, Chinese Academy of Sciences, Beijing 100101, China
[4]College of Life Sciences, University of Chinese Academy of Sciences, Beijing 100049, China
*Correspondence: lerongrong@tongji.edu.cn (R.L.), lank0305@126.com (L.K.), gaoshaorong@tongji.edu.cn (S.G.)
https://doi.org/10.1016/j.stemcr.2017.09.012

SUMMARY

Mammalian oocytes possess fascinating unknown factors, which can reprogram terminally differentiated germ cells or somatic cells into totipotent embryos. Here, we demonstrate that oocyte-specific homeobox 1 (*Obox1*), an oocyte-specific factor, can markedly enhance the generation of induced pluripotent stem cells (iPSCs) from mouse fibroblasts in a proliferation-independent manner and can replace *Sox2* to achieve pluripotency. Overexpression of *Obox1* can greatly promote mesenchymal-to-epithelial transition (MET) at early stage of OSKM-induced reprogramming, and meanwhile, the hyperproliferation of THY1-positive cells can be significantly mitigated. Subsequently, the proportion of THY1-negative cells and *Oct4*-GFP-positive cells increased dramatically. Further analysis of gene expression and targets of *Obox1* during reprogramming indicates that the expression of *Obox1* can promote epithelial gene expression and modulate cell-cycle-related gene expression. Taken together, we conclude that the oocyte-specific factor *Obox1* serves as a strong activator for somatic cell reprogramming through promoting the MET and mitigating cell hyperproliferation.

INTRODUCTION

Terminally differentiated somatic cells can be reprogrammed to become pluripotent either by somatic cell nuclear transfer (SCNT) (Gurdon et al., 1958; Wilmut et al., 1997) or by the forced expression of reprogramming factors, *Oct4* (O), *Sox2* (S), *Klf4* (K), and *c-Myc* (M) (Takahashi et al., 2007; Takahashi and Yamanaka, 2006) to generate induced pluripotent stem cells (iPSCs). Benefits by technical simplification and free of ethical concerns, iPSCs make a significant step forward for patient-specific stem cells and individualized treatment. At the same time, the iPSC generation process is more likely a stochastic event, resulting in very low efficiency (<1%) while being time-consuming (2–3 weeks) and highly dependent on cell proliferation (Kawamura et al., 2009; Li et al., 2009; Ruiz et al., 2011; Utikal et al., 2009). On the other hand SCNT, whereby a somatic nucleus is reprogrammed by oocyte cytosolic factors in a deterministic manner, is rapid, relatively efficient, and cell division independent (Jullien et al., 2011, 2014). The different efficiency between SCNT and iPSC technology (Le et al., 2014) implies that some magical factors present in the oocyte might be able to promote iPSC induction. In fact, growing evidence suggests that some oocyte-specific factors can enhance the efficiency and quality of iPSC reprogramming (Gaspar-Maia et al., 2013; Huynh et al., 2016; Jiang et al., 2013; Khaw et al., 2015; Kunitomi et al., 2016; Maekawa et al., 2011; Shinagawa et al., 2014; Singhal et al., 2010). However, although many transcription factors have been shown to enhance the generation of iPSCs, the majority of oocyte factors remain poorly investigated.

To investigate the role of oocyte factors in cellular reprogramming, we selected several highly expressed factors in oocytes based on our previously reported mass spectrometry-identified oocyte protein composition pool (Wang et al., 2010) and RNA sequencing (RNA-seq) data (Liu et al., 2016). In the present study, we focused on the maternal factor *Obox1* because it is an extremely poorly studied oocyte-specific factor in development and somatic cell reprogramming. There are eight members in the *Obox* family, six of which were reported to express in germ cells specifically (Rajkovic et al., 2002). *Obox1* was found exclusively expressed in mouse oocytes as early as one-layer follicles and throughout folliculogenesis (Rajkovic et al., 2002). In mouse stem cells, *Obox* genes were negatively regulated by *Lin28* (Park et al., 2012). CPEB, a sequence-specific RNA binding protein, binds to *Obox1* mRNA and may regulate its polyadenylation-induced translation (Racki and Richter, 2006). Recently, it was reported that *Setd1b* can promote the expression of the major oocyte transcription factors

This is an open access article under the CC BY license (http://creativecommons.org/licenses/by/4.0/).

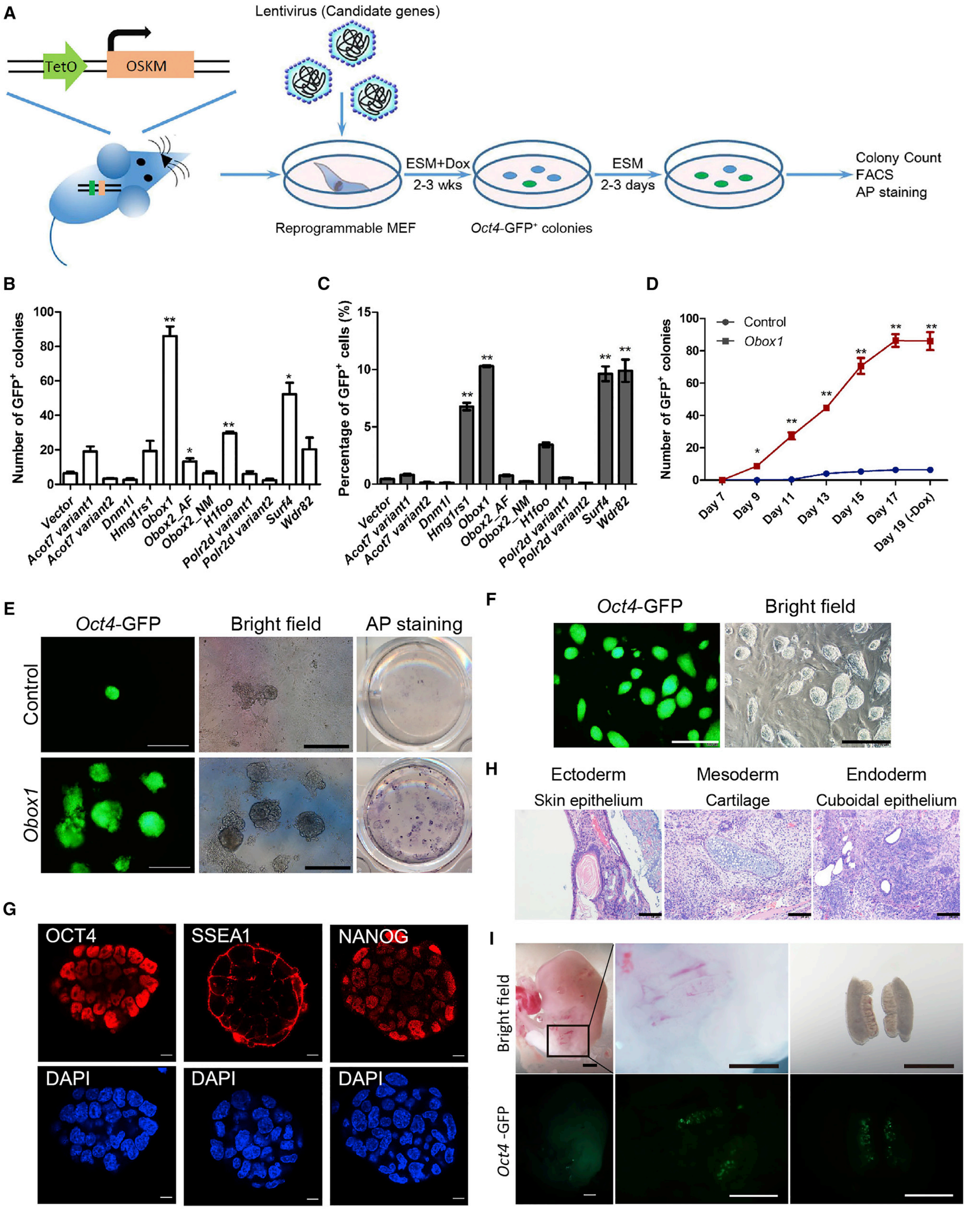

(legend on next page)

including *Obox1*, *2*, *5*, and *7* (Brici et al., 2017). However, the function of *Obox1* remains unknown, especially in embryo development and somatic cell reprogramming. Here, we show that the overexpression of *Obox1* can significantly promote the generation of iPSCs together with OSKM and can even replace *Sox2* to achieve pluripotency. Further molecular analysis indicated that the overexpression of *Obox1* can promote mesenchymal-to-epithelial transition (MET) and mitigate cell hyperproliferation, which can in turn selectively increase the proportion of THY1^{-} cells dramatically in the early stage of somatic cell reprogramming.

RESULTS

Obox1 Can Facilitate iPSC Induction

During the induction of iPSCs from somatic cells using transcription factors, only a very small proportion of cells can be reprogrammed successfully. In contrast, oocyte-based reprogramming is considered more efficient and synchronous. Recently, it has been shown that some oocyte-derived factors can indeed enhance the efficiency and quality of iPSC induction (Gonzalez-Munoz et al., 2014; Jiang et al., 2013; Khaw et al., 2015; Kunitomi et al., 2016; Maekawa et al., 2011; Shinagawa et al., 2014). We also found several highly expressed factors in oocytes in our previous study (Wang et al., 2010), after which we aimed to illustrate their roles in somatic reprogramming. To this end, we utilized reprogrammable mouse embryonic fibroblasts (MEFs) derived from the transgenic mice carrying the tetO-OSKM transgene and *Oct4*-GFP/*Rosa26*-M2rtTA (Carey et al., 2010). The induced expression of O, S, K, and M under the addition of doxycycline (Dox) was able to reprogram the MEFs into *Oct4*-GFP^{+} iPSCs (Figure 1A). We found that *Obox1*, *Surf4*, *H1foo*, *Wdr82*, and *Hmg1rs1* can facilitate somatic cell reprogramming to various extent, as judged by *Oct4*-GFP^{+} colony numbers and the percentage of *Oct4*-GFP^{+} cells (Figures 1B and 1C). Among these factors, *Obox1* exhibited the most dramatic positive effect on iPSC generation. *Obox1* was exclusively expressed in oocytes and early embryos before the 2-cell stage (Figure S1A). Overexpression of *Obox1* accelerated the formation of *Oct4*-GFP^{+} colonies and resulted in a 13-fold increase of *Oct4*-GFP^{+} colony numbers (Figures 1B and 1D). Notably, the percentage of *Oct4*-GFP^{+} cells also increased up to 10% at day 17 by exogenous *Obox1* along with OSKM (Figure 1C). The alkaline phosphatase-positive (AP^{+}) colonies were also multiplied (Figure 1E, right panel). The OSKM + *Obox1*-iPSCs exhibited typical embryonic stem cell (ESC) morphology (Figures 1E [left and middle panel], 1F, and S1B) with a compact appearance and a well-defined border and normal karyotype (Figure S1C). Quantitative real-time PCR (qRT-PCR) and immunofluorescence staining indicated that OSKM + *Obox1*-iPSCs exhibited expression of pluripotent genes at the mRNA and protein levels comparable with that of ESCs (Figures S1D and 1G). We then conducted *in vitro* and *in vivo* differentiation assays to examine the differentiation potential of OSKM + *Obox1*-iPSCs (Figures S1E, 1H, and 1I). Through embryoid body (EB)-mediated *in vitro* differentiation, the differentiated cells showed an upregulation of markers of three germ layers (Figure S1E). Teratomas also formed after subcutaneous injection of OSKM + *Obox1*-iPSCs into nude mice, with tissues of three germ layers, including skin epithelium (ectoderm), cartilage (mesoderm), and cuboidal epithelium (endoderm) (Figure 1H). Furthermore, the chimera formation assay was performed, whereby the OSKM + *Obox1*-iPS cell lines could integrate into the gonads of the chimeric mice (Figure 1I). *Obox1* is specifically expressed in rodents, and we further investigated whether mouse *Obox1* can promote human iPSC

Figure 1. Exogenous Expression of *Obox1* Promotes iPSC Generation
(A) Strategy for functional studies of candidate genes in reprogramming. Parallel experiments were performed using individual candidate genes and the empty vector as a control.
(B) The number of *Oct4*-GFP^{+} colonies was counted at day 19 after induction.
(C) The percentage of *Oct4*-GFP^{+} cells was analyzed by FACS at day 19 after induction.
(D) Kinetics of the *Oct4*-GFP^{+} colonies formation are facilitated by *Obox1*.
(E) Morphology of *Oct4*-GFP^{+} primary colonies (left and middle panels). Representative AP-stained plates are shown 19 days after induction (right panel). Scale bars, 400 μm.
(F) Morphology of OSKM + *Obox1*-iPSC lines. Scale bars, 400 μm.
(G) Immunostaining of pluripotent marker genes OCT4 (red), SSEA1 (red), and NANOG (red) in OSKM + *Obox1*-iPSC lines. Nuclear staining by DAPI (blue). Scale bars, 50 μm.
(H) H&E staining of teratoma generated from OSKM + *Obox1*-iPSCs showing representative ectodermal (epidermis), mesodermal (cartilage), and endodermal (cuboidal epithelium) tissues. Scale bars, 100 μm.
(I) Representative photos of the contribution and spatial distribution of *Oct4*-GFP^{+} cells in the gonads from E12.5 OSKM + *Obox1*-iPSC-derived chimera embryo. Scale bars, 1 mm.
Data are presented as the mean ± SEM (n = 3); *p < 0.05, **p < 0.01 by Student's t test for comparison and empty vector as control. See also Figure S1 and Table S1.

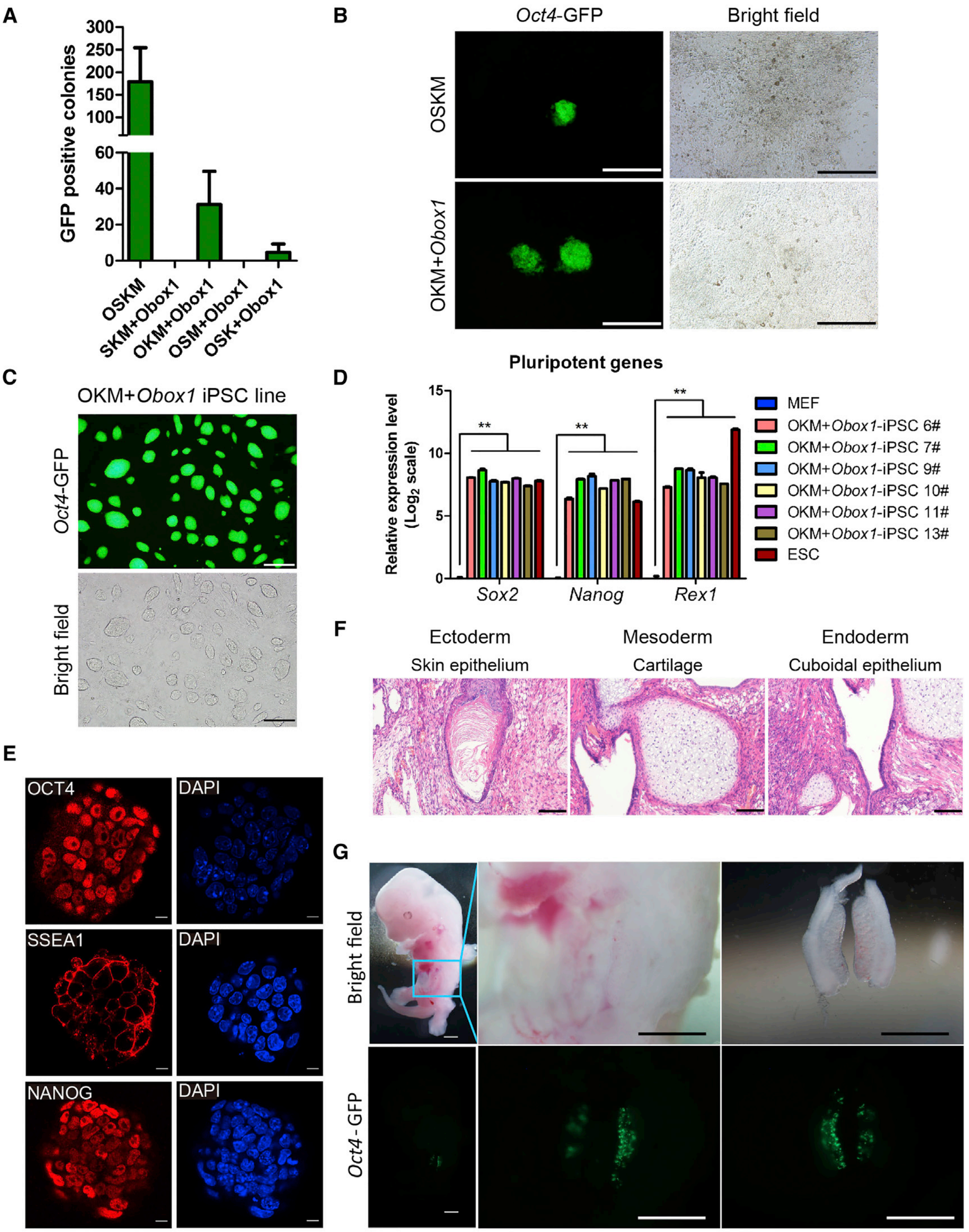

Figure 2. *Obox1* Can Replace *Sox2* during Somatic Cell Reprogramming

(A) The number of *Oct4*-GFP^{+} colonies was counted at the end of induction.

(B) Morphology of primary colonies. Scale bars, 400 μm.

(C) Morphology of OKM + *Obox1*-iPSC lines. Scale bars, 400 μm.

(D) qRT-PCR analysis shows pluripotency gene expression in OKM + *Obox1*-iPSCs. Relative mRNA expression was normalized to hypoxanthine-guanine phosphoribosyltransferase (*Hprt*) mRNA and represented relative to expression in MEFs.

(E) Immunostaining of pluripotent markers OCT4 (red), SSEA1 (red), and NANOG (red) in OKM + *Obox1*-iPSC lines. Nuclear staining by DAPI (blue). Scale bars, 50 μm.

(legend continued on next page)

induction, and no positive effects could be observed (data not shown).

Obox1 Can Replace *Sox2* to Accomplish Successful Somatic Cell Reprogramming

The drastic enhancement of somatic cell reprogramming by *Obox1* promoted us to further explore whether *Obox1* can replace Yamanaka factors. We then substituted individual factors with *Obox1* and evaluated reprogramming efficiency using the MEFs derived from transgenic mice carrying *Oct4*-GFP/*Rosa26*-M2rtTA (OG2-MEF). Finally, we found that *Obox1* can replace *Sox2* to yield *Oct4*-GFP$^+$ colonies with typical ESC colony morphology (Figures 2A and 2B). The OKM + *Obox1*-iPSCs exhibited typical ESC morphology (Figure 2C) and positive AP activity (Figure S2A). Besides, they expressed pluripotent markers and cell-surface markers of mouse ESCs (Figures 2D and 2E). Karyotype analysis showed that OKM + *Obox1*-iPSCs maintained the normal 40 chromosomes (Figure S2B). Moreover, OKM + *Obox1*-iPSCs also exhibited differentiation ability both *in vitro* and *in vivo*. EBs were formed using OKM + *Obox1*-iPSCs, and marker genes of the three germ layers were detected in the plated EBs (Figure S2C). Teratomas with three germ layers could be generated by OKM + *Obox1*-iPSCs (Figure 2F). Additionally, chimeric mice could be generated by germline transmission (Figure 2G). To understand the mechanism underlying the substitution, we collected the samples on day 3 of the reprogramming process and performed RNA-seq. Compared with OKM + empty vector, the OSKM and OKM + *Obox1* possessed differential expression genes (DEGs) (fold change >2) sharing similar pathways by gene ontology (GO) analysis (Figures S2D and S2E). Thus, oocyte factor *Obox1* not only facilitated iPSC induction, but was also able to replace the Yamanaka factor *Sox2* to accomplish the reprogramming process.

Obox1 Mitigates Cell Hyper-proliferation and Functions at the Early Stage of Reprogramming

The positive role of *Obox1* on reprogramming and its ability to replace *Sox2* prompted us to investigate the influence of *Obox1* overexpression on the activation of reprogramming factors and pluripotent genes. Although *Oct4*, *Rex1*, and *Nanog* were expressed remarkably higher under the ectopic expression of *Obox1* in reprogramming compared with the control group (Figure 3A), overexpression of *Obox1* alone could hardly activate the expression of these genes in MEFs (Figure 3A). Instead, we noticed that the cell proliferation under *Obox1* overexpression was dramatically different from that of the control. The growth curve during reprogramming showed that the overexpression of *Obox1* resulted in significant reduction of cell proliferation rate as early as the first 2 days (Figure 3B). Then we analyzed the intermediate population progression at successive time points during reprogramming. Flow cytometry showed that addition of *Obox1* promoted the transition from the THY1$^+$ cell population to the THY1$^-$ cell population (Figure 3C), which is accepted as one of the prerequisite characteristics of successful reprogramming in the early stage. Furthermore, SSEA1$^+$ and subsequent *Oct4*-GFP$^+$ cell numbers were significantly increased by forced expression of *Obox1* (Figures 3D and 1D). We then investigated the time window during which *Obox1* can enhance somatic cell reprogramming. We introduced *Obox1* at different time points during the somatic cell reprogramming process and examined the number of *Oct4*-GFP$^+$ colonies and the percentage of *Oct4*-GFP$^+$ cells at the end of reprogramming (Figures 3E–3G). Early introduction of exogenous *Obox1* before day 4 significantly increased the reprogramming efficiency while *Obox1* overexpression after day 7 showed no obvious effect. These results suggested that *Obox1* plays an important role in promoting somatic cell reprogramming at the initiation phase of reprogramming.

Genome-wide Analysis of the Effects of *Obox1* Overexpression on Somatic Reprogramming

To understand how *Obox1* promotes the reprogramming process, we employed the reprogrammable system with or without *Obox1* overexpression, and performed RNA-seq and chromatin immunoprecipitation sequencing (ChIP-seq) on day 3. Unsupervised hierarchical clustering analysis (Figure 4A) and principal component analysis (Figure S3A) based on the RNA-seq data showed high similarity among replicates of the same treatment, while the MEF sample was highly distinguishable from other samples, regardless of whether the exogenous *Obox1* was introduced or not (Figures 4A and S3A). Under the overexpression of *Obox1*, the number of DEGs (fold change $\geq$1.5 and false discovery rate $\leq$0.05) compared with the MEF was much more than that in the empty vector (control) (Figure 4B). ChIP-seq exhibited a significantly enriched binding of *Obox1* at promoters (Figure S3B). Moreover, 36.7% of the DEGs had *Obox1* binding within 2 kb of their transcription

(F) H&E staining of teratoma generated from OKM + *Obox1*-iPSCs showing representative ectodermal (epidermis), mesodermal (cartilage), and endodermal (cuboidal epithelium) tissues. Scale bars, 100 μm.
(G) Representative photos of the contribution and spatial distribution of *Oct4*-GFP$^+$ cells in the gonads from E12.5 OKM + *Obox1*-iPSC-derived chimera embryo. Scale bars, 1 mm.
Data are presented as the mean ± SEM (n = 3); **p < 0.01 by Student's t test. See also Figure S2.

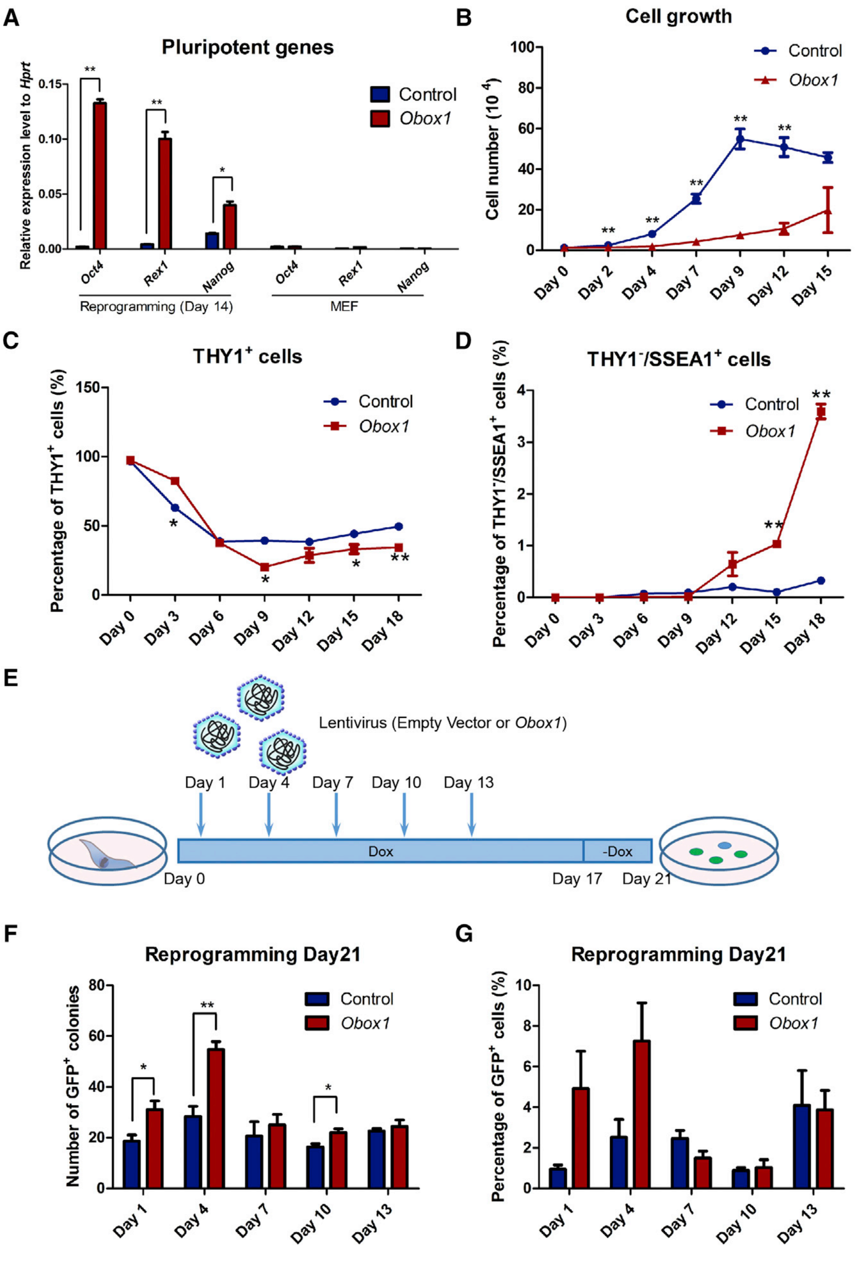

Figure 3. *Obox1* Mitigates Cell Hyper-proliferation and Functions at the Early Stage of Reprogramming

(A) qRT-PCR analysis shows pluripotent gene expression in reprogrammable cells on day 14 post induction with or without *Obox1* or *Obox1*-infected MEFs on day 4, and the empty vector-infected cells as control for each group.

(B) Proliferation curves of reprogrammable cells transduced with or without *Obox1*.

(C) Kinetic changes of percentage of THY1$^+$ population at indicated time points during reprogramming by FACS analysis.

(D) Percentage of kinetic changes of percentage of THY1$^-$/SSEA1$^+$ population during reprogramming by FACS analysis.

(E) Strategy of time-course study of *Obox1* during reprogramming.

(F) Reprogrammable cells were infected with *Obox1* at indicated time points and the number of *Oct4*-GFP$^+$ colonies were counted at 21 days post induction.

(G) Reprogrammable cells were infected with *Obox1* at indicated time points and *Oct4*-GFP$^+$ cells were analyzed by FACS at 21 days post induction.

Data are presented as the mean ± SEM (n = 3); *$p < 0.05$, **$p < 0.01$ by Student's t test for comparison and empty vector at indicated time points as control.

start sites (Figure 4C, left column). Most *Obox1* targeted genes were also the targets of *Sox2* by comparing our *Obox1* ChIP-seq dataset and the previously reported *Sox2* ChIP-seq data (Figures S3C and S3D) (Chronis et al., 2017), which provides another explanation for the ability of *Obox1* to replace *Sox2* in reprogramming.

According to the differential expression patterns in the three samples, we obtained several major gene clusters (Figures 4C and S3E; Table S2). A large number of genes increased in the early stage of reprogramming were further upregulated by *Obox1* (cluster I; ~300 genes), and GO analysis showed that they were mainly involved in epithelial cell differentiation and intermediate filament organization. Among these genes, 119 of them possess *Obox1* binding sites. Another impressive cluster (cluster II) was composed of the genes that were significantly increased in the control group compared with MEF, but strikingly decreased under *Obox1* overexpression; around half of them have *Obox1* binding on their promoters, and the pathways associated with the cell cycle and DNA replication were highly enriched in this cluster. There was a similar case in cluster III, where genes had greatly increased control than in the *Obox1* overexpression group, and genes were found to be enriched during M phase and microtubule

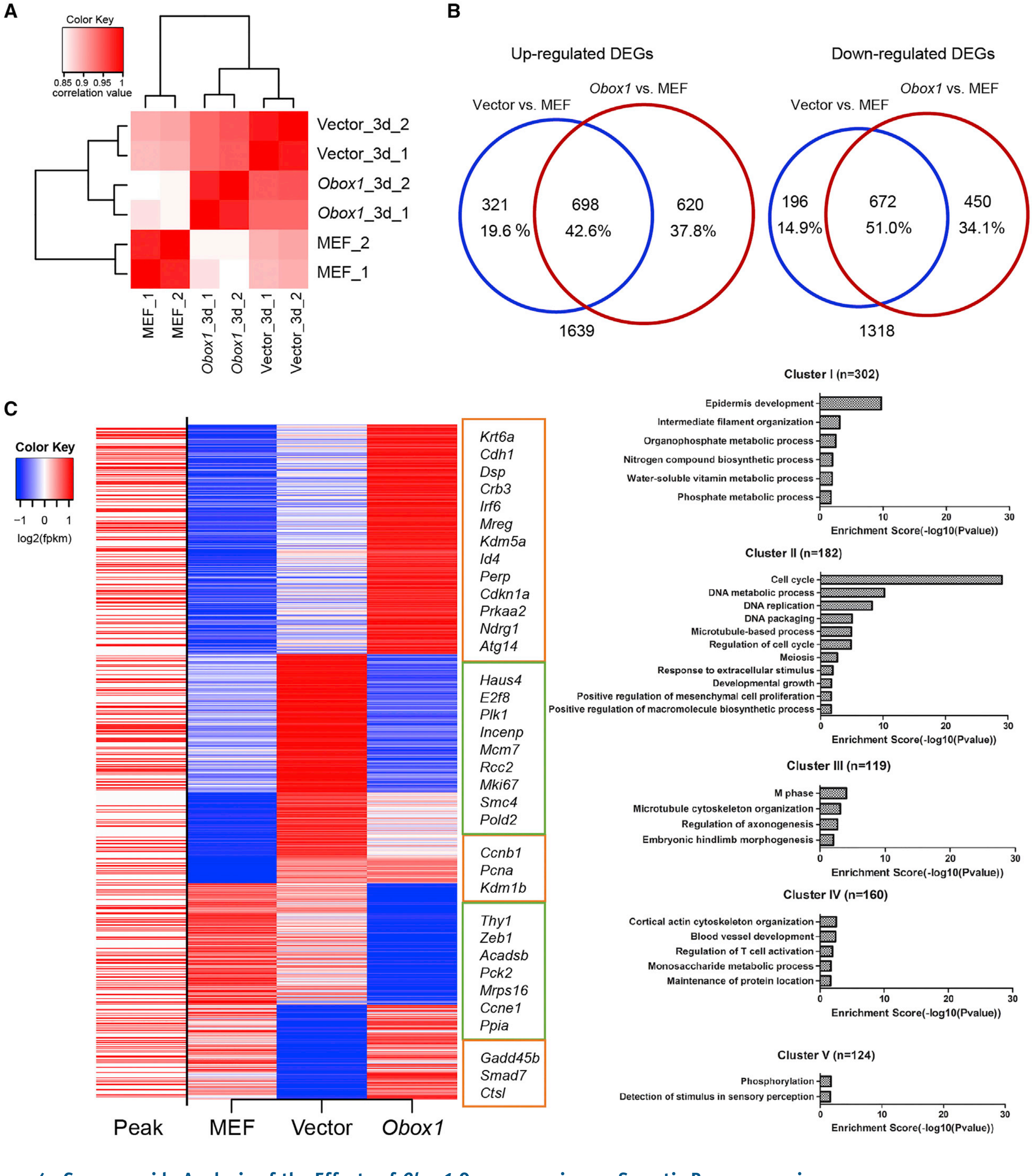

Figure 4. Genome-wide Analysis of the Effects of *Obox1* Overexpression on Somatic Reprogramming

(A) Heatmap shows the Pearson correlation of gene transcription profiles of MEFs, reprogrammable cells transduced with OSKM or OSKM plus *Obox1* on day 3 post induction.

(B) Venn diagram shows the overlap of different expression genes between samples (Vector and *Obox1*) on reprogramming day 3 and MEFs. Different expression genes were calculated by EdgeR (see Experimental Procedures).

(C) Different expression genes between samples (Vector and *Obox1*) on reprogramming day 3 were grouped into five clusters by K-means clustering based on RNA-seq data. Heatmap shows different expression genes (red, upregulated genes; blue, downregulated genes), which are targeted by *Obox1* through ChIP-seq (left column). Gene ontology analysis of each cluster is shown in the right column.

See also Figure S3 and Table S2.

cytoskeleton organization. Such a retraction in cell division-associated pathways was consistent with the slowdown in cell proliferation rate by *Obox1* overexpression in reprogramming. Those genes (cluster IV, Figure 4C) that were downregulated early in reprogramming were dramatically decreased under *Obox1* overexpression, including *Thy1* and *Zeb1*, among others. Target analysis by integration of transcriptome and ChIP-seq data with BETA showed that *Obox1* more likely acts as a repressor for these genes (Figure S3F).

Obox1 Promotes MET in the Initiation Stage of Reprogramming

The MET was identified as a hallmark at the initiation phase of MEF reprogramming (Li et al., 2010; Samavarchi-Tehrani et al., 2010). To further explore the role of *Obox1* on MET, we checked the levels of the key epithelial and mesenchymal regulators and fibroblast markers in our RNA-seq dataset. The low expression level of the epithelial genes was found in MEFs and in the empty vector control groups, while *Obox1* overexpression lead to significant upregulation of these genes, including *Cdh1*, *Ocln*, *Epcam*, and *Crb3* (Figure S4 and Table S3). Consistent with the activation of epithelial-like markers, the expression level of mesenchymal regulators, such as *Snail*, *Slug*, *Zeb1*, and *Zeb2*, were markedly downregulated in the *Obox1* group, and the fibroblast markers *Cdh2* and *Thy1* were also significantly downregulated (Figure S4). These findings suggest that *Obox1* can efficiently stimulate MET at the initiation stage of MEF reprogramming. To confirm this, we examined the RNA expression level of epithelial- and mesenchymal-associated genes during reprogramming by qRT-PCR. As shown in Figure 5A, concomitant with the Dox induction, the upregulation of epithelial regulators such as *E-cadherin*, *Dsp*, *Crb3*, and *Ocln* can be further elevated by *Obox1* from day 2 to day 6, compared with the control at indicated time points, while the expression level of mesenchymal-associated genes, such as *Twist*, *Zeb1*, and *Zeb2*, were significantly downregulated (Figure 5B). Western blot analysis further validated the increased expression of E-CADHERIN and CYTOKERATINS (Figure 5C). Prominent *Obox1* peaks in the promoter regions of epithelial-associated regulators *Dsp*, *Crb3*, and *Irf6* suggested direct binding and regulation (Figure 5D). Therefore, *Obox1* significantly promotes MET, which may in turn contribute to its role in enhancing somatic cell reprogramming.

Obox1 Mitigates Cell Hyper-proliferation by Modulating Cell-Cycle-Related Gene Expression

As mentioned above, OSKM reprogramming cells displayed a much higher proliferation rate compared with that of OSKM + *Obox1* reprogramming cells (Figure 3B). Transcriptome profile showed that the genes associated with cell-cycle regulation remained unchanged or downregulated in the *Obox1* group while these genes were markedly upregulated in the control group (Figure 4C). As shown in Figure S5A, the cyclin-dependent kinases (CDKs), cell division cycle proteins, and other cell-cycle-related genes were dramatically upregulated in OSKM reprogramming cells compared with those of OSKM + *Obox1* reprogramming cells (Figure S5A and Table S4). Next, we performed qRT-PCR to validate expression levels of the genes associated with the cell cycle during reprogramming (Figure 6A). In addition, ChIP-seq analysis showed that *Obox1* was enriched at the promoter regions of these genes, indicating direct regulation of the cell cycle by *Obox1* (Figure S5B). More interestingly, overexpression of *Obox1* alone does not affect cell number (Figure 6B), which suggests that the slowdown in cell proliferation rate by *Obox1* might depend on OSKM expression. Furthermore, the impact of *Obox1* on cell proliferation during reprogramming was also reflected in the cell-cycle properties. Distinct cell-cycle stage composition is reconstructed at the initial phase in OSKM-mediated reprogramming. Hoechst 33342 DNA staining assay and bromodeoxyuridine (BrdU) incorporation assay showed a markedly shortened G_1 phase in OSKM reprogramming cells while this shortened G_1 was not obvious in *Obox1* + OSKM reprogramming cells (Figures 6C and S5C).

Since *Obox1* accelerated the reduction in the THY1$^+$ cell population (Figure 3C), we next asked whether *Obox1* has a different effect on THY1$^+$ and THY1$^-$ cell growth. After Dox induction for 2 days, we sorted the THY1$^+$ and THY1$^-$ cells and performed a cell proliferation assay. Consistent with previous studies, OSKM induced a strong increase of cell proliferation rate in both the THY1$^+$ and THY1$^-$ cell populations during reprogramming (Figure 6D). As shown in Figure 6D, in the OSKM + *Obox1* group THY1$^-$ cells displayed increased proliferation similarly to OSKM-induced reprogramming, whereas the growth of THY1$^+$ cells was dramatically reduced and eventually ceased. This result was consistent with the expression features of cell-cycle-related genes. Most cell-cycle-related genes were downregulated by *Obox1* in both THY1$^+$ and THY1$^-$ populations, but seemed much more obvious in the THY1$^+$ population (Figure 6E).

Taken together, our data suggested that *Obox1* can modulate the expression of genes associated with the cell cycle and that a proportion of THY1$^-$ cells shows selective growth advantage over THY1$^+$ cells.

DISCUSSION

Our study aims to discover more native oocyte-derived factors that can facilitate somatic cell reprogramming

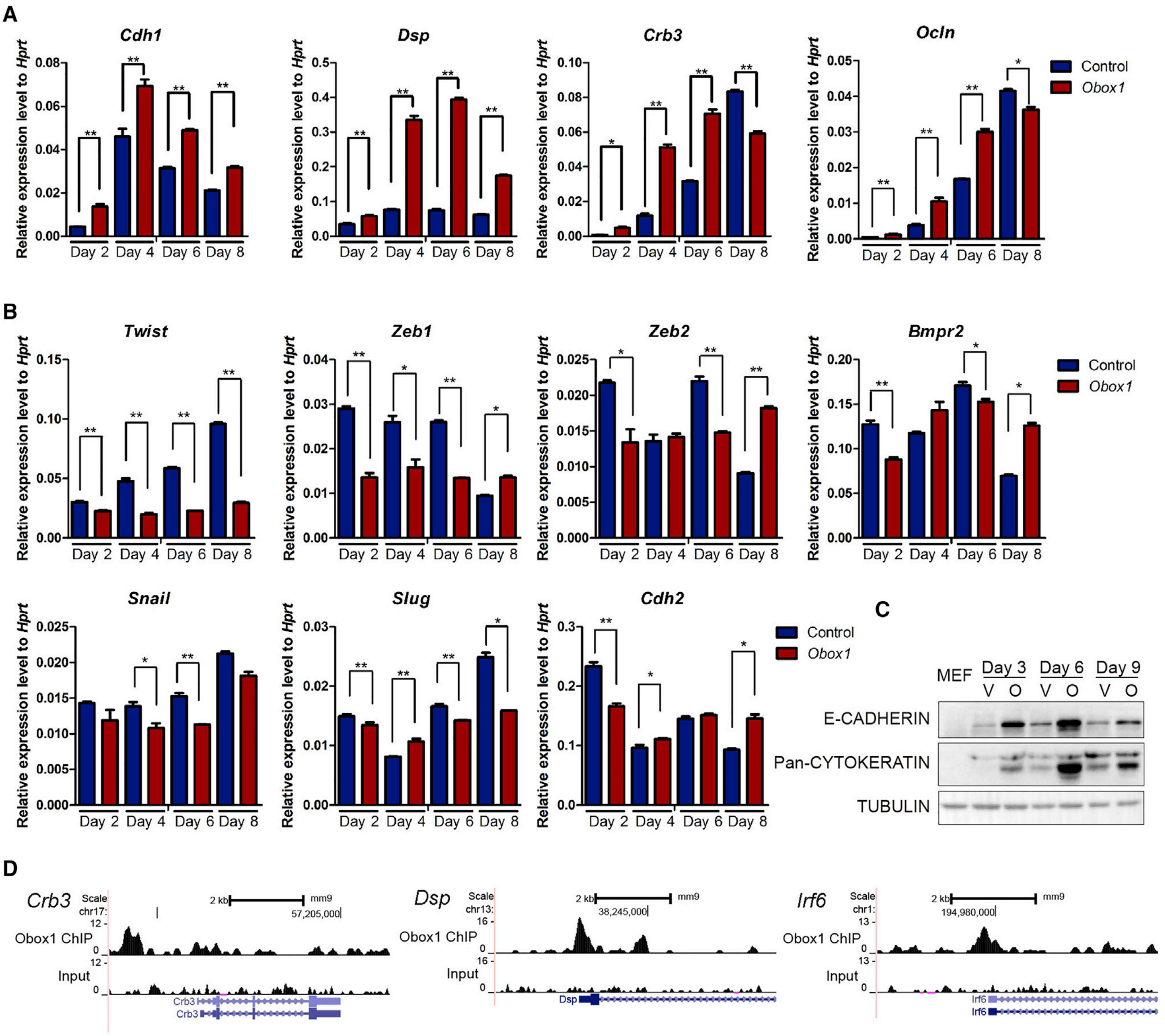

Figure 5. *Obox1* Promotes MET at the Initiation Stage of Reprogramming

(A) qRT-PCR analysis of epithelial genes expression in *Obox1*-infected reprogrammable cells at indicated time points. The expression levels were normalized to *Hprt*.

(B) qRT-PCR analysis of mesenchymal genes expression in *Obox1*-infected reprogrammable cells at indicated time points. The expression levels were normalized to *Hprt*.

(C) Western blot analysis the protein levels of E-CADHERIN and Pan-CYTOKERATIN during reprogramming; α-TUBULIN was used as a loading control (V, vector; O, *Obox1*).

(D) ChIP density profiles of *Obox1* at epithelial-related gene promoters.

Data are presented as the mean ± SEM (n = 3); *p < 0.05, **p < 0.01 by Student's t test for comparison and empty vector at indicated time points as control. See also Figure S4 and Table S3.

(Gurdon and Melton, 2008). By performing small-scale screening, we demonstrated that the oocyte factor *Obox1* can markedly facilitate iPSC induction together with OSKM and can even replace *Sox2* to accomplish somatic cell reprogramming. Furthermore, we found that overexpression of *Obox1* during reprogramming can promote MET and mitigate cell hyper-proliferation, which can in turn increase the proportion of $THY1^{-}$ cells dramatically, thus benefiting the reprogramming efficiency.

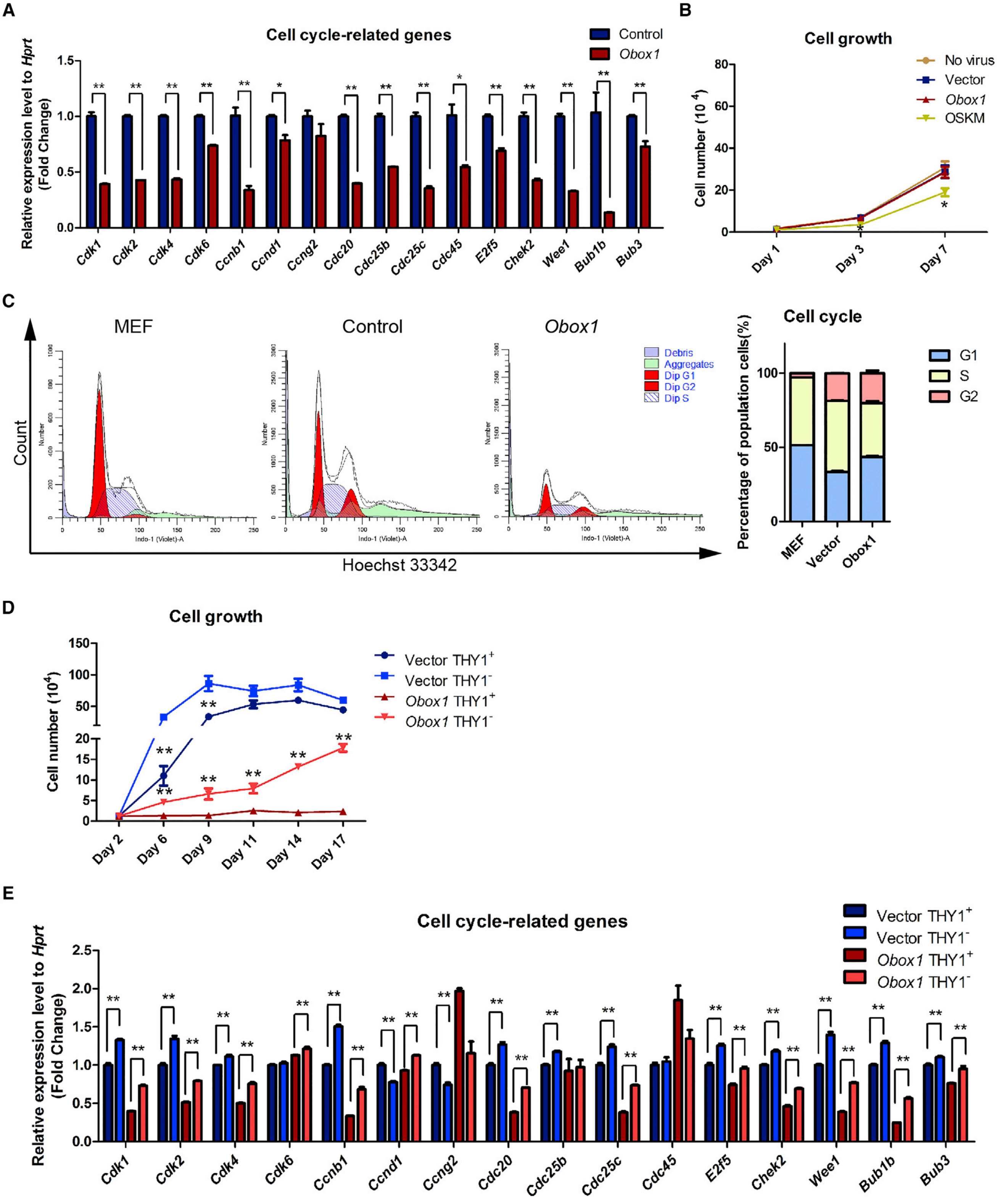

Figure 6. *Obox1* Mitigates Cell Hyper-proliferation and Enlarges the Proportion of THY1⁻ Cells during Reprogramming
(A) qRT-PCR analysis of cell-cycle-related gene expression in reprogrammable cells transduced with OSKM or OSKM plus *Obox1* on day 2 post induction. The expression level was normalized to *Hprt*.

(legend continued on next page)

As reported previously, MET is one of the critical early events in reprogramming of MEFs into iPSCs (Li et al., 2010; Samavarchi-Tehrani et al., 2010). As our ChIP-seq and RNA-seq analyses of OSKM + *Obox1* in the reprogramming showed, *Obox1* upregulated the epithelial-associated genes such as *Dsp* and *Crb3*, as well as *Smad7*, which is a key repressor of transforming growth factor β (TGF-β) signaling. TGF-β is a strong signal for EMT and the inhibition of this pathway (by small-molecule compounds or *Smad7* overexpression) have been proved able to enhance reprogramming and replace c-*Myc* and *Sox2* (Ichida et al., 2009; Li et al., 2010; Maherali and Hochedlinger, 2009). Targeting *Smad7* and the epithelial factors and subsequently promoting MET in early reprogramming by overexpression of *Obox1* may be the explanation for its ability to replace the role of *Sox2* to achieve reprogramming together with OKM.

Cell proliferation is another important characteristic in reprogramming. While some factors advanced the iPSC derivation by increased cell growth, others facilitated the reprogramming independent of proliferation (Hanna et al., 2009). Until now, the role of cell proliferation on reprogramming has remained controversial. It is reported that rapid proliferation facilitates reprogramming (Hong et al., 2009; Kawamura et al., 2009; Ruiz et al., 2011), but excessive proliferation also decreased the reprogramming efficiency of both mouse and human fibroblasts (Gupta et al., 2015; Xu et al., 2013), while inhibition of cell proliferation by chemotherapeutic drugs at an early reprogramming stage could help to improve efficiency (Xu et al., 2013). In our study an appropriate dose of ectopic *Obox1* negatively regulated cell proliferation, including downregulating cyclins, CDKs, and the genes involved in DNA replication, chromatin condensation, and cytokinesis, and upregulating cyclin-dependent kinase inhibitors. Moreover, the effect of *Obox1* was shown to be much greater on the hyper-proliferation of $THY1^+$ cells, leading to an increased proportion of $THY1^-$ cells thus able to facilitate the reprogramming process.

It is predicted that *Obox1* may function as a repressor rather than an activator (Figure S3F). This could be due to its homeodomain-related and helix-turn-helix motif, lambda-like repressor domains (Gallardo et al., 2007). Although *Obox1* and *Obox2* share 97% similarity (only six different amino acid residues are different), their effects on reprogramming efficiency were different (Figures 1B and 1C). The individual characteristics of the side chain lead to the different elaborate three-dimensional structure between *Obox1* and *Obox2*, which endows them with unique functions. For instance, the amino acid Leu76 in *Obox1*, located near the first helix, may fold in a hydrophobic pocket together with other amino acids, while Pro76 in *Obox2* may cause a turn and change the direction of the main chain of the protein (Harrison and Aggarwal, 1990; Luscombe et al., 2001; Schofield, 1987).

Obox1 is an oocyte-specific protein first induced in early follicle growth that maintains high expression in oocytes and early embryo until the 2-cell stage (Gallardo et al., 2007; Rajkovic et al., 2002), which is also supported by our study. *Obox1* belongs to the *Obox* family, which possesses the conserved homeodomain (and hence potential transcription factors) preferentially expressed in germ cells (Rajkovic et al., 2002). Knockdown of *Obox4* in oocytes resulted in abnormal metaphase I arrest via STAT3 and MPF/MAPK signaling pathways (Lee et al., 2010, 2016). *Obox6* knockout mice undergo normal early embryonic development and are fertile (Cheng et al., 2007). The functional study of *Obox1* is thus far blank. The reduction of cell proliferation rate by *Obox1* in somatic cell reprogramming mimics the early embryo state to some extent. In fact, nearly half of the DEGs specifically upregulated by *Obox1* (288/620) in our data are also highly expressed in zygote/2-cell embryo (Macfarlan et al., 2012). Taken together, this evidence suggests that *Obox1* is an extremely important factor in embryo development and reprogramming, and the functional exploration of its role during the folliculogenesis, oogenesis, and early embryo development would be very helpful in unveiling the mysterious oocyte factors and reprogramming process.

EXPERIMENTAL PROCEDURES

Candidate Genes' Lentiviral Vector Construction and iPSC Derivation

Full-length mouse *Acot7* (NM_001146057, NM_133348), *Dnm1l* (NM_152816.3), *H1foo* (NM_138311), *Hmga1-rs1*

(B) Proliferation curves of MEFs transduced with or without *Obox1* or OSKM, respectively (empty vector as control).
(C) Cell-cycle analysis by FACS in MEFs or the reprogramming cells on day 2 post infection and comparison of the indicated cell-cycle phase (empty vector versus MEF; Obox1 versus empty vector).
(D) Proliferation curve of $THY1^+$ and $THY1^-$ cells sorted from reprogrammable cells on day 2 post induction. The cells were seeded in 24-well plates at a density of 1.2×10^4 cells per well and counted at indicated time points, and the $THY1^+$ population and $THY1^-$ population compared in the same group.
(E) qRT-PCR analysis of cell-cycle-related gene expression in $THY1^+$ and $THY1^-$ cell populations sorted from reprogrammable cells on day 2 post induction. The expression levels were normalized to *Hprt*. $THY1^+$ in vector or *Obox1* group as control.
Data are presented as the mean ± SEM (n = 3); $^*p < 0.05$, $^{**}p < 0.01$ by Student's t test for comparison. See also Figure S5 and Table S4.

(NM_001166476), *Polr2d* (NM_027002, NM_027101), *Surf4* (NM_011512), *Wdr82* (NM_029896), *Obox1* or FLAG-tagged-*Obox1* (NM_027802), and *Obox2* (NM_145708, AF461107) were cloned and inserted into the FUW-TET-On vector. Inducible iPSCs were generated as previously described (Brambrink et al., 2008; Stadtfeld et al., 2008). ESC-like colonies appeared 2–3 weeks after induction. The colonies were picked and propagated after Dox withdrawal.

The analyses of iPSCs, such as qRT-PCR, AP staining, *in vitro* differentiation, teratoma formation, and chimera experiments, were performed as previously described (Kang et al., 2009), as detailed in the Supplemental Experimental Procedures. All primer sequences are available in Table S1.

RNA Sequencing

Total RNA from independent biological replicates of each uninduced MEF, induced MEFs with or without *Obox1* at 72 hr for OSKM + *Obox1* system, and of overexpressing OSKM, OKM + *Obox1*, or OKM + empty vector at 72 hr for replacement system, was isolated using the QIAGEN RNeasy Kit (Germantown, USA). The RNA samples were subject to mRNA fragmentation, cDNA synthesis, and library preparation using a KAPA Stranded RNA-Seq Kit Illumina platform (KK8440; Kapa, Wilmington, USA). All adapters were diluted from the adapters offered by TruSeq Library Prep Pooling kit (Illumina, USA). Single-end 50-bp sequencing was further performed on HiSeq 2500 (Illumina) at Berry Genomics.

ChIP Sequencing

ChIP experiments were performed using the MAGnify Chromatin Immunoprecipitation System (Invitrogen, Carlsbad, USA) according to the manufacturer's recommendations. Ten million cells on reprogramming day 3 were used for per immunoprecipitation reaction. In brief, cells were chemically crosslinked at room temperature by the addition of formaldehyde to 1% final concentration for 10 min and quenched with 0.125 M final concentration glycine. Crosslinked cells were resuspended in lysis buffer and chromatin was sonicated to 100–400 bp with a Covaris M220 system. The sonicated chromatin was then immunoprecipitated with 3 μg of FLAG antibody (Sigma F1804; St. Louis, USA) for each immunoprecipitation reaction. A fraction of "whole-cell extract" obtained without antibody was retained as an input control. DNA was eluted by elution buffer and purified through phenol-chloroform extraction and isopropanol precipitation. The sequence libraries were generated using a KAPA HyperPlus Library Preparation kit (KK8510; Kapa), following the manufacturer's instructions. Single-end 50-bp sequencing was further performed on a HiSeq 2500 (Illumina) at Berry Genomics.

Trimming and Alignment of Sequencing Reads

All of the RNA-seq reads were first mapped to hg19 reference genome using TopHat (v 2.1.1) with default parameters (Trapnell et al., 2009). Gene expression for each sample was quantified to FPKM (fragments per kilobase of transcript per million mapped reads) using Cufflinks (v 2.2.1) to eliminate the effects of sequencing depth and transcript length (Trapnell et al., 2010). All ChIP-seq samples were mapped to mm9 reference genome using the bowtie 2 (v 2.2.9) command with default parameters (Li and Durbin, 2009).

Cell Growth Curve, BrdU Assay, and Cell-Cycle Analysis

For growth curve analysis, the cells were plated onto 12-well plates at a density of 1.2×10^4 cells per well, and were harvested every 48 or 72 hr and counted in a cell-counting chamber. Each group contained two or three replicates. For BrdU incorporation assay, cells were treated with BrdU for 60 min. The cells were stained following the manufacturer's instructions (FITC BrdU Flow Kit, Becton Dickinson, San Jose, USA). For cell-cycle analysis, cells were stained with Hoechst 33342 (Sigma) and analyzed using a Beckmam Coulter CytoFLEX S flow cytometer or BD FACSAria II (for sorting).

Mice

All of our study procedures were consistent with those in the Tongji University guide for the care and use of laboratory animals.

Statistical Analysis

Results are presented as the mean ± SEM of independent experiments. Significance was determined by Student's t test.

ACCESSION NUMBERS

The sequencing datasets have been deposited in NCBI's Gene Expression Omnibus and are accessible through the accession number GEO: GSE97859.

SUPPLEMENTAL INFORMATION

Supplemental Information includes Supplemental Experimental Procedures, five figures, and four tables and can be found with this article online at https://doi.org/10.1016/j.stemcr.2017.09.012.

AUTHOR CONTRIBUTIONS

L.W. designed and performed the experiments, data analysis, discussion, and writing; Y.W. performed bioinformatics analysis; B.P., Z.H., Y.D., K.C., M.G., H.L., X.C., X.K., Y.Z., Y.B., Y.W., and H.W. contributed to experimental work and discussion; S.G., L.K., and R.L. supervised the study and contributed to writing.

ACKNOWLEDGMENT

We would like to thank Yang Shi from the Yong Zhang laboratory for help with the bioinformatics analysis and Professor Jiuhong Kang from Tongji University for providing the E-CADHERIN antibodies. We are also grateful to our laboratory colleagues, especially Dr. Jiayu Chen, for their assistance with experiments and advice. This work was supported by the National Natural Science Foundation of China (31430056, 31501183, 31371512, 81322029, and 31721003), the Ministry of Science and Technology of China (2016YFA0100400), the Shanghai Subject Chief Scientist Program (15XD1503500), and the Chenguang Program (15CG19) from the Shanghai Education Development Foundation and the Shanghai Municipal Education Commission.

Received: April 18, 2017
Revised: September 14, 2017
Accepted: September 15, 2017
Published: October 12, 2017

REFERENCES

Brambrink, T., Foreman, R., Welstead, G.G., Lengner, C.J., Wernig, M., Suh, H., and Jaenisch, R. (2008). Sequential expression of pluripotency markers during direct reprogramming of mouse somatic cells. Cell Stem Cell *2*, 151–159.

Brici, D., Zhang, Q., Reinhardt, S., Dahl, A., Hartmann, H., Schmidt, K., Goveas, N., Huang, J., Gahurova, L., Kelsey, G., et al. (2017). Setd1b, encoding a histone 3 lysine 4 methyltransferase, is a maternal effect gene required for the oogenic gene expression program. Development *144*, 2606–2617.

Carey, B.W., Markoulaki, S., Beard, C., Hanna, J., and Jaenisch, R. (2010). Single-gene transgenic mouse strains for reprogramming adult somatic cells. Nat. Methods *7*, 56–59.

Cheng, W.C., Hsieh-Li, H.M., Yeh, Y.J., and Li, H. (2007). Mice lacking the Obox6 homeobox gene undergo normal early embryonic development and are fertile. Dev. Dyn. *236*, 2636–2642.

Chronis, C., Fiziev, P., Papp, B., Butz, S., Bonora, G., Sabri, S., Ernst, J., and Plath, K. (2017). Cooperative binding of transcription factors orchestrates reprogramming. Cell *168*, 442–459.e20.

Gallardo, T.D., John, G.B., Shirley, L., Contreras, C.M., Akbay, E.A., Haynie, J.M., Ward, S.E., Shidler, M.J., and Castrillon, D.H. (2007). Genomewide discovery and classification of candidate ovarian fertility genes in the mouse. Genetics *177*, 179–194.

Gaspar-Maia, A., Qadeer, Z.A., Hasson, D., Ratnakumar, K., Leu, N.A., Leroy, G., Liu, S., Costanzi, C., Valle-Garcia, D., Schaniel, C., et al. (2013). MacroH2A histone variants act as a barrier upon reprogramming towards pluripotency. Nat. Commun. *4*, 1565.

Gonzalez-Munoz, E., Arboleda-Estudillo, Y., Otu, H.H., and Cibelli, J.B. (2014). Cell reprogramming. Histone chaperone ASF1A is required for maintenance of pluripotency and cellular reprogramming. Science *345*, 822–825.

Gupta, M.K., Teo, A.K.K., Rao, T.N., and Bhatt, S. (2015). Excessive cellular proliferation negatively impacts reprogramming efficiency of human fibroblasts. Stem Cells Transl. Med. *4*, 1101–1108.

Gurdon, J.B., Elsdale, T.R., and Fischberg, M. (1958). Sexually mature individuals of *Xenopus laevis* from the transplantation of single somatic nuclei. Nature *182*, 64–65.

Gurdon, J.B., and Melton, D.A. (2008). Nuclear reprogramming in cells.pdf. Science *322*, 5.

Hanna, J., Saha, K., Pando, B., van Zon, J., Lengner, C.J., Creyghton, M.P., van Oudenaarden, A., and Jaenisch, R. (2009). Direct cell reprogramming is a stochastic process amenable to acceleration. Nature *462*, 595–601.

Harrison, S.C., and Aggarwal, A.K. (1990). DNA recognition by proteins with the helix-turn-helix motif. Annu. Rev. Biochem. *59*, 933–969.

Hong, H., Takahashi, K., Ichisaka, T., Aoi, T., Kanagawa, O., Nakagawa, M., Okita, K., and Yamanaka, S. (2009). Suppression of induced pluripotent stem cell generation by the p53-p21 pathway. Nature *460*, 1132–1135.

Huynh, L.M., Shinagawa, T., and Ishii, S. (2016). Two histone variants TH2A and TH2B enhance human induced pluripotent stem cell generation. Stem Cells Dev. *25*, 251–258.

Ichida, J.K., Blanchard, J., Lam, K., Son, E.Y., Chung, J.E., Egli, D., Loh, K.M., Carter, A.C., Di Giorgio, F.P., Koszka, K., et al. (2009). A small-molecule inhibitor of tgf-Beta signaling replaces sox2 in reprogramming by inducing nanog. Cell Stem Cell *5*, 491–503.

Jiang, J., Lv, W., Ye, X., Wang, L., Zhang, M., Yang, H., Okuka, M., Zhou, C., Zhang, X., Liu, L., et al. (2013). Zscan4 promotes genomic stability during reprogramming and dramatically improves the quality of iPS cells as demonstrated by tetraploid complementation. Cell Res. *23*, 92–106.

Jullien, J., Pasque, V., Halley-Stott, R.P., Miyamoto, K., and Gurdon, J.B. (2011). Mechanisms of nuclear reprogramming by eggs and oocytes: a deterministic process? Nat. Rev. Mol. Cell Biol. *12*, 453–459.

Jullien, J., Miyamoto, K., Pasque, V., Allen, G.E., Bradshaw, C.R., Garrett, N.J., Halley-Stott, R.P., Kimura, H., Ohsumi, K., and Gurdon, J.B. (2014). Hierarchical molecular events driven by oocyte-specific factors lead to rapid and extensive reprogramming. Mol. Cell *55*, 524–536.

Kang, L., Wang, J., Zhang, Y., Kou, Z., and Gao, S. (2009). iPS cells can support full-term development of tetraploid blastocyst-complemented embryos. Cell Stem Cell *5*, 135–138.

Kawamura, T., Suzuki, J., Wang, Y.V., Menendez, S., Morera, L.B., Raya, A., Wahl, G.M., and Izpisua Belmonte, J.C. (2009). Linking the p53 tumour suppressor pathway to somatic cell reprogramming. Nature *460*, 1140–1144.

Khaw, S.L., Min-Wen, C., Koh, C.G., Lim, B., and Shyh-Chang, N. (2015). Oocyte factors suppress mitochondrial polynucleotide phosphorylase to remodel the metabolome and enhance reprogramming. Cell Rep. *12*, 1080–1088.

Kunitomi, A., Yuasa, S., Sugiyama, F., Saito, Y., Seki, T., Kusumoto, D., Kashimura, S., Takei, M., Tohyama, S., Hashimoto, H., et al. (2016). H1foo has a pivotal role in qualifying induced pluripotent stem cells. Stem Cell Reports *6*, 825–833.

Le, R., Kou, Z., Jiang, Y., Li, M., Huang, B., Liu, W., Li, H., Kou, X., He, W., Rudolph, K.L., et al. (2014). Enhanced telomere rejuvenation in pluripotent cells reprogrammed via nuclear transfer relative to induced pluripotent stem cells. Cell Stem Cell *14*, 27–39.

Lee, H.S., Kim, E.Y., Kim, K.H., Moon, J., Park, K.S., Kim, K.S., and Lee, K.A. (2010). Obox4 critically regulates cAMP-dependent meiotic arrest and MI-MII transition in oocytes. FASEB J. *24*, 2314–2324.

Lee, H.S., Kim, K.H., Kim, E.Y., Lee, S.Y., Ko, J.J., and Lee, K.A. (2016). Obox4-silencing-activated STAT3 and MPF/MAPK signaling accelerate nuclear membrane breakdown in mouse oocytes. Reproduction *151*, 369–378.

Li, H., and Durbin, R. (2009). Fast and accurate short read alignment with Burrows-Wheeler transform. Bioinformatics *25*, 1754–1760.

Li, H., Collado, M., Villasante, A., Strati, K., Ortega, S., Canamero, M., Blasco, M.A., and Serrano, M. (2009). The Ink4/Arf locus is a barrier for iPS cell reprogramming. Nature *460*, 1136–1139.

Li, R., Liang, J., Ni, S., Zhou, T., Qing, X., Li, H., He, W., Chen, J., Li, F., Zhuang, Q., et al. (2010). A mesenchymal-to-epithelial transition initiates and is required for the nuclear reprogramming of mouse fibroblasts. Cell Stem Cell *7*, 51–63.

Liu, X., Wang, C., Liu, W., Li, J., Li, C., Kou, X., Chen, J., Zhao, Y., Gao, H., Wang, H., et al. (2016). Distinct features of H3K4me3 and H3K27me3 chromatin domains in pre-implantation embryos. Nature *537*, 558–562.

Luscombe, N.M., Laskowski, R.A., and Thornton, J.M. (2001). Amino acid–base interactions a three-dimensional analysis of protein–DNA interactions at an atomic level.pdf. Nucleic Acids Res. *29*, 15.

Macfarlan, T.S., Gifford, W.D., Driscoll, S., Lettieri, K., Rowe, H.M., Bonanomi, D., Firth, A., Singer, O., Trono, D., and Pfaff, S.L. (2012). Embryonic stem cell potency fluctuates with endogenous retrovirus activity. Nature *487*, 57–63.

Maekawa, M., Yamaguchi, K., Nakamura, T., Shibukawa, R., Kodanaka, I., Ichisaka, T., Kawamura, Y., Mochizuki, H., Goshima, N., and Yamanaka, S. (2011). Direct reprogramming of somatic cells is promoted by maternal transcription factor Glis1. Nature *474*, 225–229.

Maherali, N., and Hochedlinger, K. (2009). Tgfbeta signal inhibition cooperates in the induction of iPSCs and replaces Sox2 and cMyc. Curr. Biol. *19*, 1718–1723.

Park, G.T., Seo, Y.M., Lee, S.Y., and Lee, K.A. (2012). Lin28 regulates the expression of neuropeptide Y receptors and oocyte-specific homeobox genes in mouse embryonic stem cells. Clin. Exp. Reprod. Med. *39*, 87–93.

Racki, W.J., and Richter, J.D. (2006). CPEB controls oocyte growth and follicle development in the mouse. Development *133*, 4527–4537.

Rajkovic, A., Yan, C., Yan, W., Klysik, M., and Matzuk, M.M. (2002). Obox, a family of homeobox genes preferentially expressed in germ cells. Genomics *79*, 711–717.

Ruiz, S., Panopoulos, A.D., Herrerias, A., Bissig, K.D., Lutz, M., Berggren, W.T., Verma, I.M., and Izpisua Belmonte, J.C. (2011). A high proliferation rate is required for cell reprogramming and maintenance of human embryonic stem cell identity. Curr. Biol. *21*, 45–52.

Samavarchi-Tehrani, P., Golipour, A., David, L., Sung, H.K., Beyer, T.A., Datti, A., Woltjen, K., Nagy, A., and Wrana, J.L. (2010). Functional genomics reveals a BMP-driven mesenchymal-to-epithelial transition in the initiation of somatic cell reprogramming. Cell Stem Cell *7*, 64–77.

Schofield, P.N. (1987). Patterns, puzzles and paradigms the riddle of the homeobox.pdf. Trends Neurosci. *10*, 3.

Shinagawa, T., Takagi, T., Tsukamoto, D., Tomaru, C., Huynh, L.M., Sivaraman, P., Kumarevel, T., Inoue, K., Nakato, R., Katou, Y., et al. (2014). Histone variants enriched in oocytes enhance reprogramming to induced pluripotent stem cells. Cell Stem Cell *14*, 217–227.

Singhal, N., Graumann, J., Wu, G., Araúzo-Bravo, M.J., Han, D.W., Greber, B., Gentile, L., Mann, M., and Schöler, H.R. (2010). Chromatin-remodeling components of the BAF complex facilitate reprogramming. Cell *141*, 943–955.

Stadtfeld, M., Maherali, N., Breault, D.T., and Hochedlinger, K. (2008). Defining molecular cornerstones during fibroblast to iPS cell reprogramming in mouse. Cell Stem Cell *2*, 230–240.

Takahashi, K., Tanabe, K., Ohnuki, M., Narita, M., Ichisaka, T., Tomoda, K., and Yamanaka, S. (2007). Induction of pluripotent stem cells from adult human fibroblasts by defined factors. Cell *131*, 861–872.

Takahashi, K., and Yamanaka, S. (2006). Induction of pluripotent stem cells from mouse embryonic and adult fibroblast cultures by defined factors. Cell *126*, 663–676.

Trapnell, C., Pachter, L., and Salzberg, S.L. (2009). TopHat: discovering splice junctions with RNA-Seq. Bioinformatics *25*, 1105–1111.

Trapnell, C., Williams, B.A., Pertea, G., Mortazavi, A., Kwan, G., van Baren, M.J., Salzberg, S.L., Wold, B.J., and Pachter, L. (2010). Transcript assembly and quantification by RNA-Seq reveals unannotated transcripts and isoform switching during cell differentiation. Nat. Biotechnol. *28*, 511–515.

Utikal, J., Polo, J.M., Stadtfeld, M., Maherali, N., Kulalert, W., Walsh, R.M., Khalil, A., Rheinwald, J.G., and Hochedlinger, K. (2009). Immortalization eliminates a roadblock during cellular reprogramming into iPS cells. Nature *460*, 1145–1148.

Wang, S., Kou, Z., Jing, Z., Zhang, Y., Guo, X., Dong, M., Wilmut, I., and Gao, S. (2010). Proteome of mouse oocytes at different developmental stages. Proc. Natl. Acad. Sci. USA *107*, 17639–17644.

Wilmut, I., Schnieke, A.E., McWhir, J., Kind, A.J., and Campbell, K.H. (1997). Viable offspring derived from fetal and adult mammalian cells. Nature *385*, 810–813.

Xu, Y., Wei, X., Wang, M., Zhang, R., Fu, Y., Xing, M., Hua, Q., and Xie, X. (2013). Proliferation rate of somatic cells affects reprogramming efficiency. J. Biol. Chem. *288*, 9767–9778.

Stem Cell Reports, Volume *9*

Supplemental Information

Oocyte-Specific Homeobox 1, *Obox1*, Facilitates Reprogramming by Promoting Mesenchymal-to-Epithelial Transition and Mitigating Cell Hyperproliferation

Li Wu, You Wu, Bing Peng, Zhenzhen Hou, Yu Dong, Kang Chen, Mingyue Guo, Han Li, Xia Chen, Xiaochen Kou, Yanhong Zhao, Yan Bi, Yixuan Wang, Hong Wang, Rongrong Le, Lan Kang, and Shaorong Gao

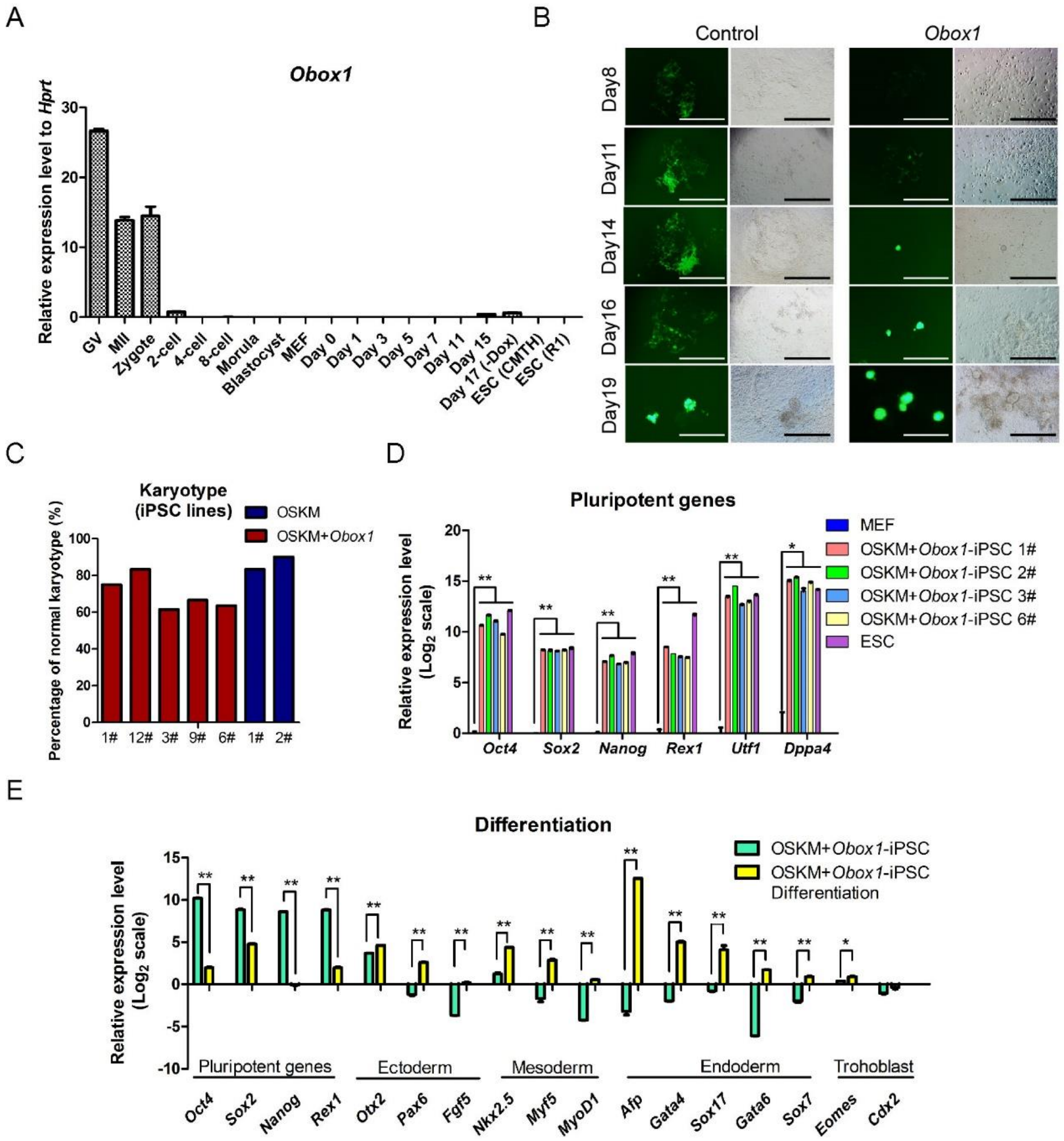

Figure S1. Exogenous Expression of *Obox1* Can Promote iPSC Generation. Related to Figure 1.

(A) Expression patterns of *Obox1* in mouse preimplantation embryos and reprogramming process by qRT-PCR analysis. Relative mRNA level was normalized to hypoxanthine-guanine phosphoribosyltransferase (*Hprt*) (MEF as control).

(B) Morphology of reprogrammable cells at indicated time points. Scale bars, 600 μm.

(C) Karyotype analysis of OSKM+*Obox1*- and OSKM-iPSC lines.

(D) qRT-PCR analysis reveals pluripotent gene expression in OSKM+*Obox1*-iPSCs. Relative mRNA expression was represented relative to expression in MEF as a control.

(E) qRT-PCR analysis shows the upregulation of markers for the three germ layers during in vitro differentiation of OSKM+*Obox1*-iPSCs. Relative mRNA expression was represented relative to expression in the undifferentiated iPSCs as a control.

Data are represented as the mean ± SEM (n = 3); * $p < 0.05$, ** $p < 0.01$ by Student's t test for comparison.

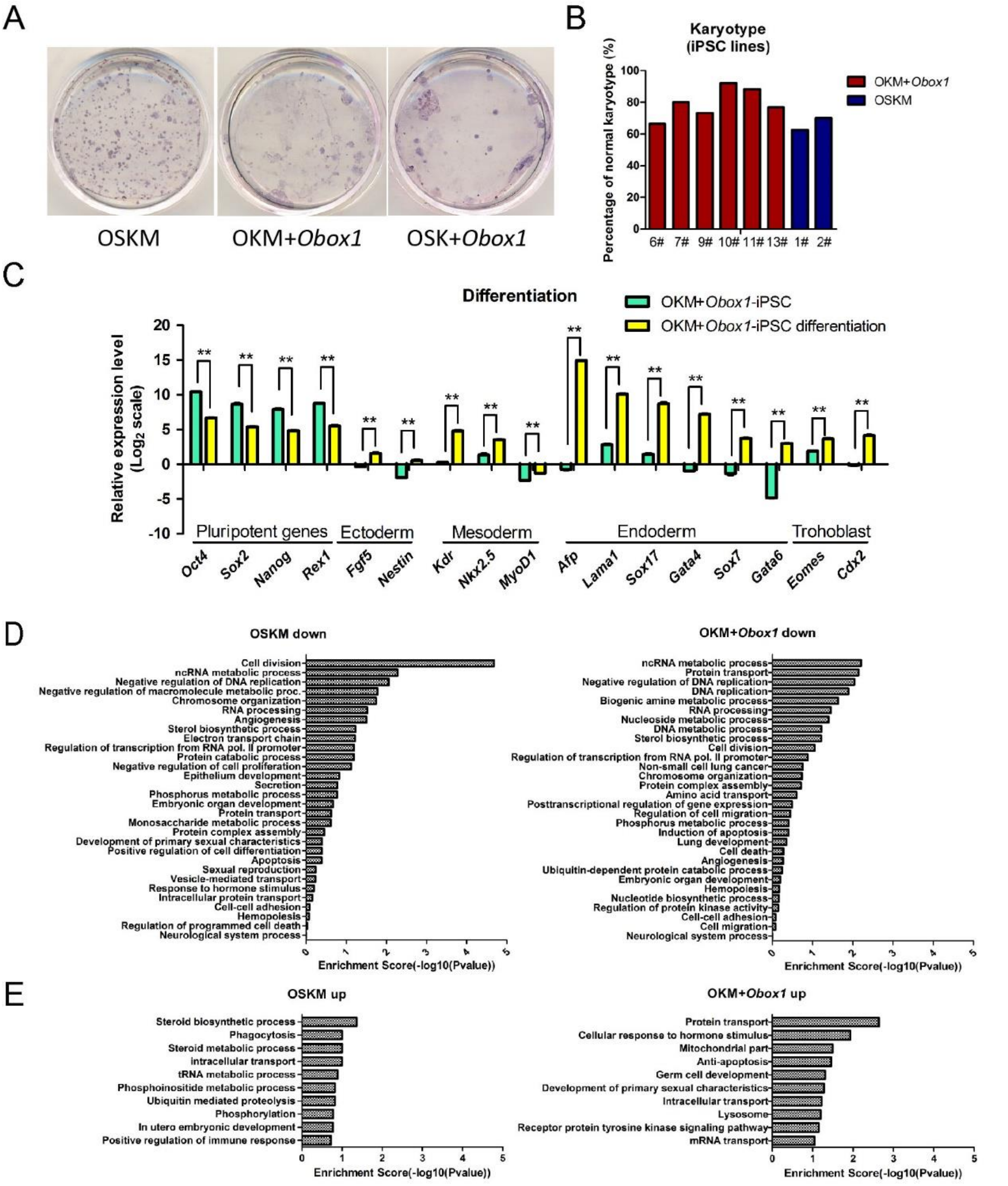

Figure S2. Characterization of OKM+*Obox1*-iPSCs and Genome-wide Analysis of OSKM and OKM+*Obox1* in Reprogramming. Related to Figure 2.

(A) The iPSCs colonies were detected by AP staining.

(B) Karyotype analysis of OSKM- and OKM+*Obox1*-iPSC lines.

(C) qRT-PCR analysis shows the upregulation of markers for the three germ layers during in vitro differentiation of OKM+*Obox1*-iPSCs. Relative mRNA expression is represented relative to expression in the undifferentiated iPSCs as a control. Data are represented as the mean ± SEM (n = 3); * $p < 0.05$, ** $p < 0.01$ by Student's t test for comparison.

(D) Gene ontology analysis of different expression genes downregulated in the samples of OSKM and OKM+*Obox1*, comparing with OKM+Vector, on reprogramming day 3.

(E) Gene ontology analysis of different expression genes upregulated in the samples of OSKM and OKM+*Obox1*, comparing with OKM+Vector, on reprogramming day 3.

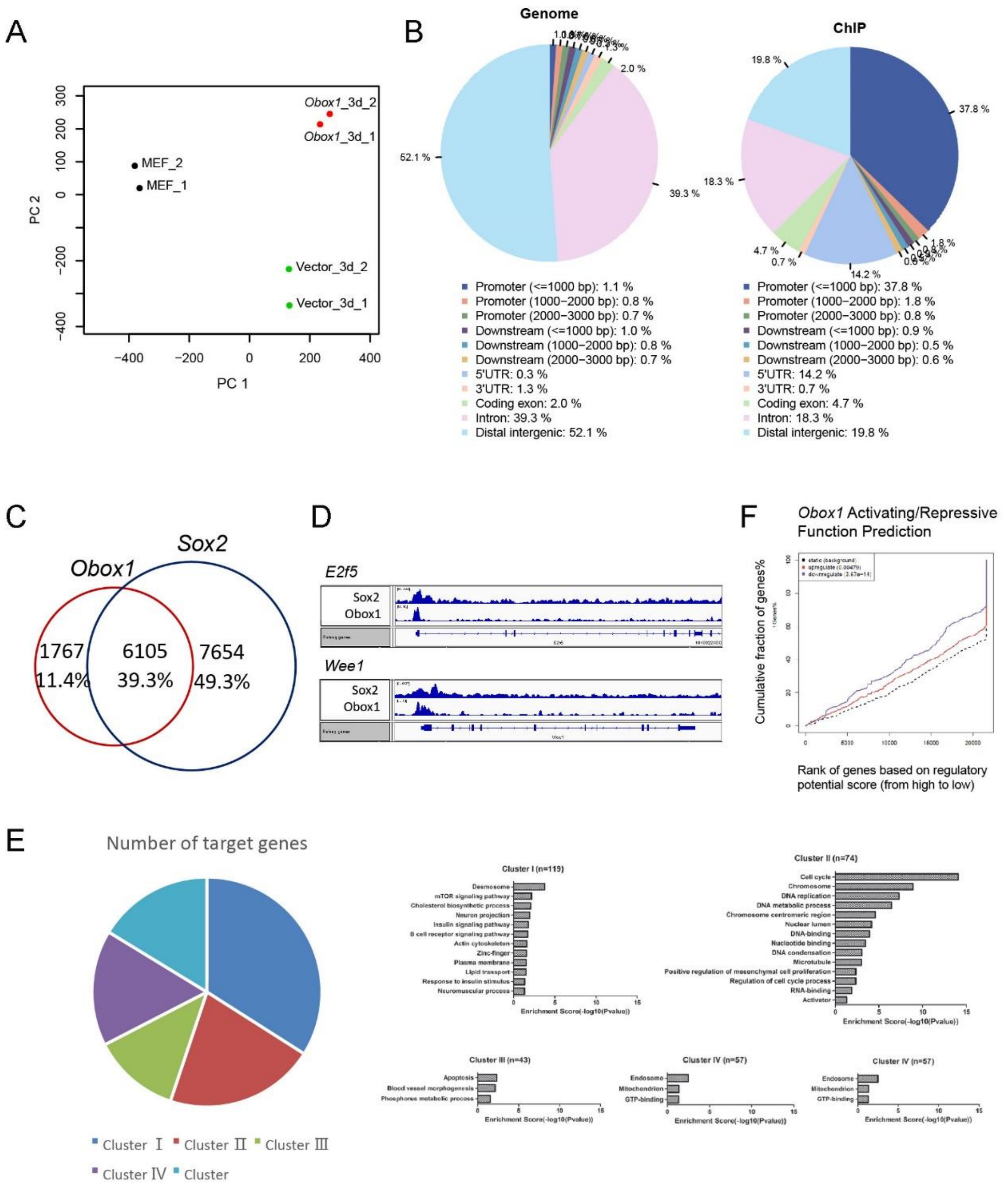

Figure S3. Genome-wide Analysis of the Effects of *Obox1* Overexpression on Somatic Reprogramming. Related to Figure 4.

(A) Principal component analysis (PCA) of global expression profiles of indicated samples. Contributions of each cell (dots) to the first two principal components (PC1 and PC2).

(B) Pie charts show genome distribution of *Obox1* binding sites on reprogramming day 3.

(C) Venn diagram shows the overlap of target genes between *Obox1* in OSKM+*Obox1* samples and *Sox2* in OSKM previously reported.

(D) ChIP density profiles of *Obox1* and *Sox2* at cell cycle-related gene promoters.

(E) Pie chart shows different expression genes between samples (*Obox1* and Vector) which are also targeted

by *Obox1* through ChIP-seq. These genes were clustered by K-means. Gene ontology analysis of each cluster was shown in the right panel.

(F) Activation or repression function prediction of *Obox1* on reprogramming day3.

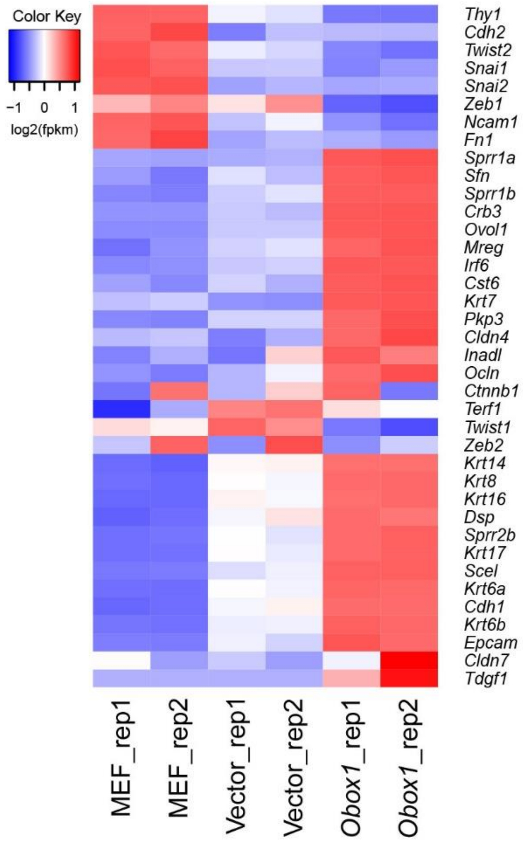

Figure S4. *Obox1* Promotes MET in the Initiation Stage of Reprogramming. Related to Figure 5. Heatmap shows the expression patterns of epithelial and mesenchymal-associated genes. FPKM values were analyzed using Cufflinks and represented in color (Red: upregulated genes; blue: downregulated genes).

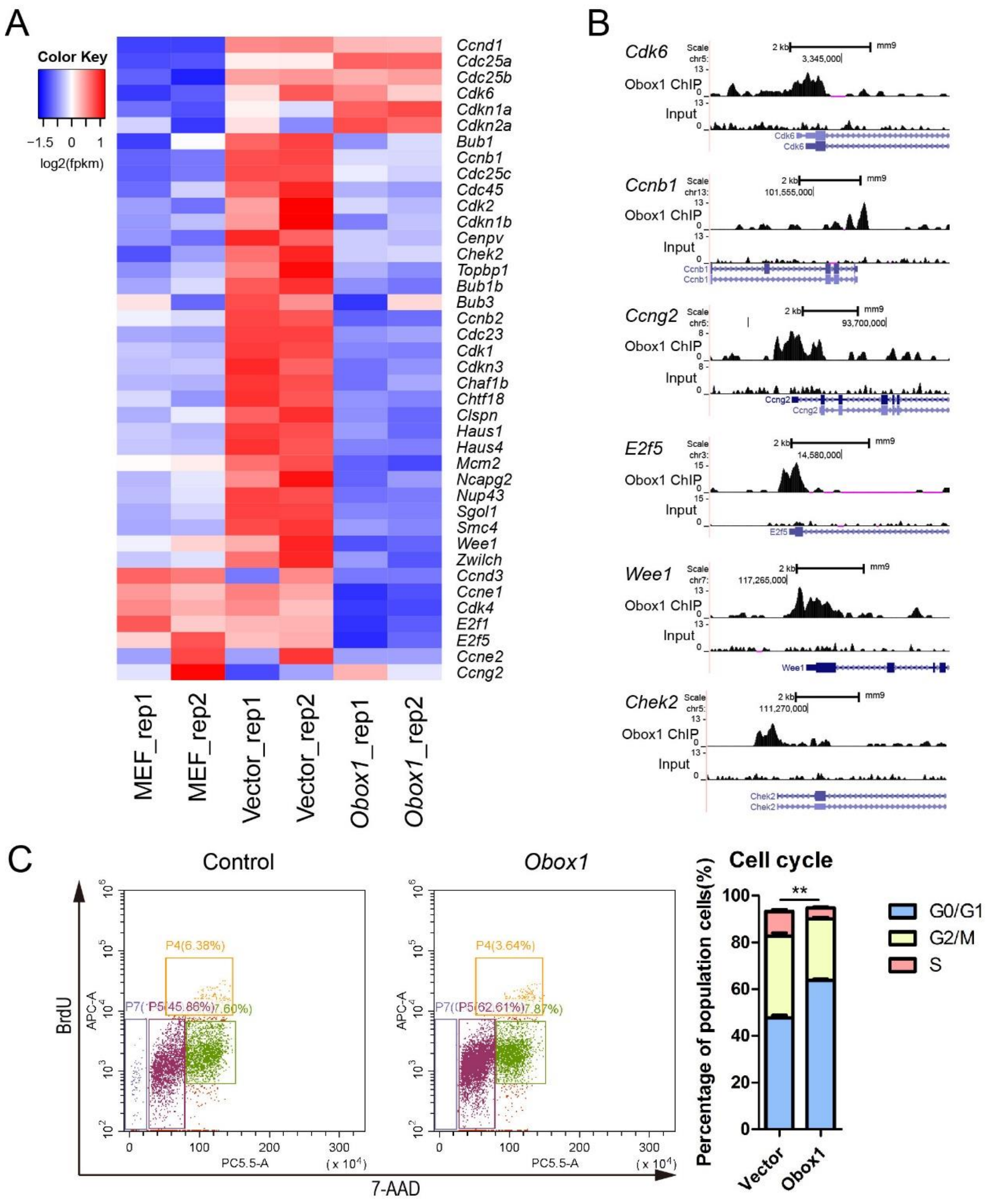

Figure S5. ***Obox1*** **Mitigates Cell Hyper-proliferation by Modulating Cell-cycle-related Genes Expression. Related to Figure 6.**

(A) Heatmap shows the expression patterns of cell cycle-related genes in the indicated samples. FPKM values were analyzed using Cufflinks and represented in color (red: upregulated genes; blue: downregulated genes).

(B) ChIP density profiles of *Obox1* at cell-cycle-related gene promoters.

(C) Cell-cycle analysis of the reprogramming cells on day 2 post-induction by FACS. Reprogrammable fibroblasts were transfected with *Obox1* or empty vector (control) and were labeled with 5-bromodeoxyuridine (BrdU) and 7-AAD after 2-days induction by Dox. Data are represented as the mean ± SEM (n = 3); $**p < 0.01$ by Student's t test for comparison and empty vector in each phase as control.

Table S1. Primer Sequences Used in this Paper. Related to Figure 1. (see attached the excel file named "Table S1 primers").

Table S2. Different Expression Genes with or without *Obox1* on Reprogramming Day 3. Related to Figure 4. (see attached the excel file named "Table S2 DEGs")

Different expression genes between samples (Vector and *Obox1*) on reprogramming day 3 were grouped into five clusters by K-means clustering based on RNA-seq data. The potential targets of *Obox1* based on the ChIP-seq data are presented as 1 in the binding column.

Table S3. Epithelial and Mesenchymal Genes Regulated by *Obox1* at Early Stage during Reprogramming in RNA-seq Data. Related to Figure 5.

Gene Name	MEF_rep1	MEF_rep2	Vector_rep1	Vector_rep2	*Obox1* _rep1	*Obox1* _rep2	km$cluster
Thy1	1.19478961	1.17627334	-0.08987027	-0.21514261	-1.02267208	-1.04337799	1
cdh2	1.1518509	1.38002649	-0.98388087	-0.48171426	-0.52271892	-0.54356335	1
Twist2	1.28280276	1.13412558	-0.13649092	-0.30950535	-0.90974834	-1.06118373	1
Snai1	1.33111583	1.19484816	-0.41882224	-0.40705972	-0.93435999	-0.76572204	1
Snai2	1.26898814	1.30970161	-0.69786155	-0.5555709	-0.67886999	-0.64638731	1
Zeb1	0.53447952	0.92126836	0.19763562	0.84001313	-1.1679552	-1.32544143	1
Ncam1	1.15524151	1.27416863	-0.44594427	-0.08301379	-0.82715139	-1.07330069	1
Fn1	1.15029952	1.41267184	-0.68570788	-0.50110428	-0.60757672	-0.76858248	1
Sprr1a	-0.66658993	-0.69949865	-0.62509236	-0.58854239	1.26027542	1.3194479	2
SFN	-0.75101009	-1.01051372	-0.24053218	-0.49281824	1.21644024	1.27843399	2
SPRR1B	-0.91067661	-0.97195562	-0.37619676	-0.20749396	1.23014658	1.23617637	2
Crb3	-0.81702459	-0.81702459	-0.4047521	-0.50795579	1.26587727	1.2808798	2
Ovol1	-0.86900487	-0.86518482	-0.39538943	-0.3939873	1.24545531	1.27811112	2
Mreg	-1.06732939	-0.82288821	-0.33864536	-0.22394931	1.15331378	1.29949849	2
Irf6	-0.90115179	-0.84664117	-0.41859092	-0.35226852	1.25736147	1.26129094	2
Cst6	-0.70792666	-0.89895809	-0.32517639	-0.60469395	1.23874204	1.29801305	2
Krt7	-0.48069164	-0.3675408	-0.83103995	-0.85513001	1.25347495	1.28092744	2
Pkp3	-0.90527369	-0.9249384	-0.32495373	-0.33006029	1.1371158	1.34811032	2
Cldn4	-0.52441729	-0.42753272	-0.97163878	-0.60581528	1.13788384	1.39152023	2
Inadl	-0.94768337	-0.60563864	-1.03283367	0.34535136	1.25911607	0.98168825	2
Ocln	-0.81537323	-0.98512755	-0.55791816	-0.09900547	1.11883026	1.33859415	2
Ctnnb1	-1.04312919	1.05639042	-0.5480357	0.37130786	1.16440302	-1.00093641	3
Terf1	-1.60502563	-0.62252579	0.92398174	1.05796195	0.25158087	-0.00597313	3
Twist1	0.25778189	0.10424331	1.15535101	0.85324054	-1.02732958	-1.34328716	3
Zeb2	-0.4248737	1.17554912	-0.86024516	1.34722358	-0.86032226	-0.37733157	3
Krt14	-1.11078979	-1.19050215	0.05862167	0.07977907	1.08104951	1.08184169	4
Krt8	-1.08468652	-1.1257301	0.01296174	-0.06162595	1.11376026	1.14532057	4
Krt16	-1.12113827	-1.12773275	0.07726782	-0.04724189	1.08269297	1.13615212	4
Dsp	-1.18967522	-1.09729687	-0.07458716	0.20257152	1.12072473	1.038263	4
Sprr2b	-1.08132986	-1.04027598	-0.00081845	-0.20301549	1.12088335	1.20455643	4
Krt17	-1.07986303	-1.07481482	0.0108102	-0.15858346	1.11405835	1.18839276	4
Scel	-1.02931643	-0.99810855	-0.25391749	-0.11317974	1.18904691	1.2054753	4
Krt6a	-1.08561696	-1.10124775	0.00079924	-0.09402318	1.15497857	1.12511009	4
CDH1	-1.1423196	-1.10212199	-0.07072346	0.09409541	1.11156943	1.10950021	4
Krt6b	-1.04165463	-1.0630391	-0.12620211	-0.11555294	1.21005579	1.13639298	4
Epcam	-0.97888062	-0.99703633	-0.1142405	-0.32499888	1.28678063	1.1283757	4
Cldn7	0.01816466	-0.73160057	-0.40121976	-0.73162276	-0.0926879	1.93896633	5
Tdgf1	-0.59747418	-0.59747418	-0.59747418	-0.59747418	0.596494	1.79340272	5

Table S4. Cell-cycle-related Genes Regulated by *Obox1* at Early Stage during Reprogramming in RNA-seq Data. Related to Figure 6.

Gene Name	MEF_rep1	MEF_rep2	Vector_rep	Vector_rep	*Obox1* _rep	*Obox1* _rep	km$cluster
Ccnd1	-1.2691359	-1.283439	0.79142408	0.82035271	0.48033135	0.46046673	1
Cdc25a	-1.2017403	-1.1448599	0.11698711	0.12444912	1.059101	1.04606293	1
Cdc25b	-1.0546181	-1.4980622	0.64615635	0.71242016	0.54419463	0.64990919	1
Cdk6	-1.337109	-1.1003249	0.20943429	1.11785049	0.7692668	0.34088234	1
Cdkn1a	-0.9616175	-1.1764443	0.09357923	-0.241192	1.06478083	1.22089378	1
Cdkn2a	-0.3055234	-1.3357983	0.21298791	-0.7819603	1.21185399	0.99844006	1
Bub1	-1.3042323	-0.0138197	1.02925382	1.27666671	-0.7405146	-0.2473539	2
Ccnb1	-1.0400265	-0.8900404	1.19416565	1.25176417	-0.2486945	-0.2671685	2
Cdc25c	-1.0758373	-0.9003883	1.22431	1.190874	-0.1235188	-0.3154395	2
Cdc45	-0.9924902	-0.3218972	1.03179774	1.46292221	-0.5194378	-0.6608947	2
Cdk2	-0.6535664	-0.9520592	0.64237871	1.71678425	-0.2706404	-0.4828971	2
Cdkn1b	-0.6979677	-0.4351792	0.67428546	1.72390639	-0.852496	-0.412549	2
Cenpv	-0.7106052	-0.9729497	1.45492351	1.04475188	-0.348588	-0.4675325	2
Chek2	-1.1594275	-0.6338279	0.9465372	1.47000592	-0.3496982	-0.2735896	2
Topbp1	-0.7556225	-0.4003039	0.80845837	1.65280036	-0.5271847	-0.7781476	2
Bub1b	-0.5937549	-0.2468363	1.10634307	1.40021768	-0.7487379	-0.9172317	3
Bub3	0.18219483	-1.0229312	1.2098031	0.73522645	-1.3603866	0.25609334	3
Ccnb2	-0.1070647	-0.2469067	1.25337942	1.13414975	-1.0531101	-0.9804477	3
Cdc23	-0.5862805	-0.6343451	1.28766304	1.29099221	-0.7382695	-0.6197601	3
Cdk1	-0.3868754	-0.4739167	1.32322433	1.21274425	-0.8157819	-0.8593946	3
Cdkn3	-0.4266378	-0.3821121	1.45402257	1.04854583	-0.9560651	-0.7377533	3
Chaf1b	-0.4897097	-0.513188	1.35615323	1.18525317	-0.9554549	-0.5830538	3
Chtf18	-0.268972	-0.7053869	1.39225698	1.13299915	-0.7236042	-0.827293	3
Clspn	-0.5502371	-0.1361782	1.05072751	1.40488646	-0.7505688	-1.0186299	3
Haus1	-0.3555721	-0.4888558	1.31865408	1.19856779	-0.6631122	-1.0096819	3
Haus4	-0.3857273	-0.4382543	1.38604776	1.13746006	-0.8476166	-0.8519095	3
Mcm2	0.01598365	0.12854553	0.9650325	1.18520867	-1.0630413	-1.231729	3
Ncapg2	-0.4728761	-0.1635253	0.76289004	1.61035993	-1.0501739	-0.6866748	3
Nup43	-0.4555852	-0.1896167	1.28132533	1.1968358	-0.9511737	-0.8817855	3
Sgol1	-0.5573422	-0.3016527	1.2766524	1.25353174	-0.8161601	-0.8550292	3
Smc4	-0.5810727	-0.504778	1.21611933	1.34981804	-0.6649751	-0.8151115	3
Wee1	-0.0992637	0.28404185	0.5510529	1.47095907	-1.1630848	-1.0437054	3
Zwilch	-0.3760704	-0.2139381	0.94812912	1.46433726	-0.6778914	-1.1445664	3
Ccnd3	1.04526873	0.89860162	-0.9097487	0.7854304	-0.9098107	-0.9097413	4
Ccne1	0.68876784	0.41789441	0.85302794	0.58969591	-1.3884482	-1.1609379	4
Cdk4	0.79575972	0.54225309	0.77790114	0.4406684	-1.3300242	-1.2265581	4
E2f1	1.12814613	0.3569501	0.48906453	0.5027206	-1.3807259	-1.0961554	4
E2f5	0.30729495	1.15058281	0.43983491	0.55217282	-1.4279329	-1.0219526	4
Ccne2	-0.6451431	1.2286046	-0.6449047	1.35143575	-0.6451425	-0.6448501	5
Ccng2	-0.1947631	1.68280367	-1.214312	-0.6070192	0.50699211	-0.1737015	5

SUPPLEMENTAL EXPERIMENTAL PROCEDURES

Mice and Cell Culture

The specific pathogen-free mice were housed in the animal facility of Tongji University. All our study procedures were consistent with the Tongji University Guide for the care and use of laboratory animals. Mouse embryonic fibroblasts (MEFs) were derived from 13.5-dpc embryos. MEFs were maintained in DMEM (Life Technologies) medium supplemented with 10% (vol/vol) FBS (Gibco) and 1 mM L-glutamine (Merck Millipore). ESCs and iPSCs were cultured on mitomycin C treated MEFs in ESM containing DMEM (Merck Millipore) supplemented with 15% (v/v) fetal bovine serum (Hyclone, Logan, UT), 1 mM L-glutamine (Merck Millipore), 0.1 mM mercaptoethanol (Merck Millipore), 1% nonessential amino acid stock (Merck Millipore), and 1000 U/mL LIF (Merck Millipore).

Induction of iPSCs

The constructed plasmid (in Fuw-TET-On vector) preparation and iPSCs induction procedure were performed according to a previously reported method (Kang et al., 2009). Plasmids were extracted with Plasmid Mini Kit (Tiangen, China) and EndoFree Plasmid Maxi Kit (Cwbio, China). HEK293T cells were transfected with the plasmids along with the lentivirus packaging plasmids psPAX2 and pMD2G (VigoFect, China). The medium containing virus was collected 48 hours after transfection. For OSKM+*Obox1* system, reprogrammable MEFs were seeded in 12-well plates at a density of 1.2×10^4 cells per well, and for OSKM or OKM+*Obox1* system, OG2-MEFs were seeded in 6-well plates at a density of 3-5×10^4 cells per well, then were infected with virus-containing medium for 10-12 hours. Infected MEFs were cultured in ESM supplemented with 1 μg/mL Dox. The cells were observed and tested at indicated time points during reprogramming. The colonies were cultured in ESM in the absence of doxycycline for 2-3 days before being mechanically picked.

Alkaline Phosphatase Staining

To detect alkaline phosphatase (AP) staining, the cytochemical assay was performed using alkaline phosphatase staining kit (Beyotime, China) according to the manufacturer's protocol. In briefly, the cells at the end of reprogramming were washed once by DPBS, and fixed by 10% formaldehyde solution for 5 minutes at room temperature. Then, the cells were washed once by deionized water and stained by the reagent provide by the kit.

FACS Analysis

Cells were trypsinized, washed twice with DPBS, resuspended in FACS buffer (PBS + 0.1% BSA) and analyzed *Oct4*-GFP$^+$ population. For analysis and/or sorting of intermediates, cells were stained with 5 μL of PE/Cy7-conjugated antibody against THY1.2 (BioLegend, 140310) and/or APC-conjugated antibody against SSEA1 (BioLegend, 125608) in 100 μL FACS buffer per 10^6 cells. After 30 minutes staining on ice, cells were washed once in FACS buffer and suspended in FACS buffer for sorting by FACS Aria II (BD) or analyzing by CytoFLEX S (Beckman Coulter).

In Vitro Differentiation

For spontaneous differentiation, iPSCs were trypsinized and a total of 5×10^4 cells per drop were cultured in hanging drop for 2 days and transferred to ultra-low cluster plates (Costar, USA) in DMEM (Gibco) supplemented with 15% (v/v) FBS, 1 mM L-glutamine (Merck Millipore), 0.1 mM mercaptoethanol (Merck Millipore), 1% nonessential amino acid stock (Merck Millipore), but without LIF. Five days later,

EBs were collected on gelatin-coated tissue cultured dishes for 21 days. Total RNA of EBs was extracted and analyzed for the markers for three embryonic germ layers using qRT-PCR. The primer sequences are available in the Table S1.

Teratoma Formation

The iPSCs were trypsinized and a total of 2-5×10^6 iPSCs were subcutaneously injected into the groin of SCID mice. The tumors were dissected 4 weeks after the injection and processed for hematoxylin-eosin staining.

Chimeric Mouse Generation

To produce chimeric mice, the appropriate number of iPSCs were trypsinized and aggregated with two ICR 8-cell stage embryos removed the zona pellucida in each pit. After a 2-day culture, the embryos were transplanted into the uterus of pseudo-pregnant mice. The *Oct4*-GFP^+ germ cells were detected in the gonads from E 12.5 embryos.

Immunofluorescence Staining

Immunofluorescence staining was performed as previously described (Gao et al., 2013). Primary antibodies used were anti-OCT4 (Santa Cruz, SC-5279), anti-NANOG (Cosmo Bio, RCAB001P), anti-SSEA1 (Millipore, MAB4301). DNA was labeled with DAPI (1 μg/mL, Merck Millipore). The following fluorochrome conjugated secondary antibodies were used: Alexa Fluor 594 donkey anti-mouse IgG (Thermo Fisher, A21203), Alexa Fluor 594 donkey anti-rabbit IgG (Thermo Fisher, A21207). The stained cells mounted on slides were observed using an LSM 880 microscope (Zeiss, Germany,) with a Plan Neofluar 63×/1.4 Oil DIC objective.

Quantitative real-time PCR (qRT-PCR)

Total RNA was extracted using TRNzol Universal Reagent (Tiangen, Beijing, China) and reverse transcribed using the 5X All-In-One RT MasterMix (ABM, Richmond, Canada). Quantitative reverse-transcription PCR was performed with SYBR®FAST Universal qPCR Kit (KAPA, Wilmington, US) and the ABI7500 Fast Real-time PCR system (Applied Biosystems, Foster City, CA). The reactions were performed in triplicate using 1/10 concentration of the cDNA obtained as described above. Relative mRNA expression is normalized to *Hprt* as an endogenous control using the ΔΔCT or ΔCT method. Primer sequences are available in the Table S1 (Gao et al., 2013; Le et al., 2014; Samavarchi-Tehrani et al., 2010; Takahashi and Yamanaka, 2006; Xiang et al., 2012; Xiong et al., 2016; Yang et al., 2016).

Oocyte Collection and Embryo Culture

Full grown germinal vesicle-intact (GV) oocytes were obtained from 6-8 weeks female C57BL/6j mice after injection of 10 IU pregnant mare serum gonadotropin (PMSG) for 48 hours, and freed of attached cumulus cells as previously described (Wang et al., 2010). Metaphase II (MII) oocytes were collected from the oviducts of the mice after consecutive injection of females at 8–10 weeks of age with PMSG and human chorionic gonadotrophin (hCG) at 44–48 hours intervals, and the adherent cumulus cells were removed by hyaluronidase treatment. The zygotes were collected from the oviducts of the mice that were mated with male mice. Embryos were cultured in CZB medium at 37 ℃ in a humidified atmosphere of 5% CO_2 in air (Gao et al., 2003). Embryos normally cleave and develop to blastocysts and were collected at different stages.

Western Blot Analysis

After removal of the feeder cells, pluripotent stem cells were washed once with DPBS and boiled to 98 ℃ for 10 minutes in loading buffer (BioRad) with 5% β-mercaptoethanol (Amersham, CT). Anti α-TUBULIN (Proteintech, 66031-1-Ig) was used as endogenous control and Anti-E-CADHERIN (Abcam, Ab11512), Pan-CYTOKERATIN (Abcam, Ab7753) was used. ECL peroxidase-labeled sheep anti-mouse antibody (GE Healthcare, NA931VS) or goat anti-rat antibody (Santa Cruz, SC-2006) were used as secondary antibodies.

SUPPLEMENTAL REFERENCES

Gao, S., Chung, Y.G., Williams, J.W., Riley, J., Moley, K., and Latham, K.E. (2003). Somatic cell-like features of cloned mouse embryos prepared with cultured myoblast nuclei. Biology of reproduction *69*, 48-56.

Gao, Y., Chen, J., Li, K., Wu, T., Huang, B., Liu, W., Kou, X., Zhang, Y., Huang, H., Jiang, Y., *et al.* (2013). Replacement of Oct4 by Tet1 during iPSC induction reveals an important role of DNA methylation and hydroxymethylation in reprogramming. Cell stem cell *12*, 453-469.

Kang, L., Wang, J., Zhang, Y., Kou, Z., and Gao, S. (2009). iPS cells can support full-term development of tetraploid blastocyst-complemented embryos. Cell stem cell *5*, 135-138.

Le, R., Kou, Z., Jiang, Y., Li, M., Huang, B., Liu, W., Li, H., Kou, X., He, W., Rudolph, K.L., *et al.* (2014). Enhanced telomere rejuvenation in pluripotent cells reprogrammed via nuclear transfer relative to induced pluripotent stem cells. Cell stem cell *14*, 27-39.

Samavarchi-Tehrani, P., Golipour, A., David, L., Sung, H.K., Beyer, T.A., Datti, A., Woltjen, K., Nagy, A., and Wrana, J.L. (2010). Functional genomics reveals a BMP-driven mesenchymal-to-epithelial transition in the initiation of somatic cell reprogramming. Cell stem cell *7*, 64-77.

Takahashi, K., and Yamanaka, S. (2006). Induction of pluripotent stem cells from mouse embryonic and adult fibroblast cultures by defined factors. Cell *126*, 663-676.

Wang, S., Kou, Z., Jing, Z., Zhang, Y., Guo, X., Dong, M., Wilmut, I., and Gao, S. (2010). Proteome of mouse oocytes at different developmental stages. Proceedings of the National Academy of Sciences of the United States of America *107*, 17639-17644.

Xiang, X., Deng, Z., Zhuang, X., Ju, S., Mu, J., Jiang, H., Zhang, L., Yan, J., Miller, D., and Zhang, H.G. (2012). Grhl2 determines the epithelial phenotype of breast cancers and promotes tumor progression. PloS one *7*, e50781.

Xiong, Z., He, W., Chen, J., Han, Z., Sun, X., and Gao, S. (2016). Study of Mouse Androgenetic Haploid Embryonic Stem Cells on Proliferation Ability. Chinese Journal of Cell Biology *38*, 9.

Yang, P., Shen, W.B., Reece, E.A., Chen, X., and Yang, P. (2016). High glucose suppresses embryonic stem cell differentiation into neural lineage cells. Biochemical and biophysical research communications *472*, 306-312.
